# Piperazine-substituted derivatives of favipiravir for Nipah virus inhibition: What do in silico studies unravel?

**DOI:** 10.1007/s42452-020-04051-9

**Published:** 2021-01-11

**Authors:** Raju Lipin, Anantha Krishnan Dhanabalan, Krishnasamy Gunasekaran, Rajadurai Vijay Solomon

**Affiliations:** 1 Department of Chemistry, Madras Christian College (Autonomous), [affiliated to University of Madras], Tambaram East, Chennai, Tamil Nadu 600 059 India; 2 CAS in Crystallography and Biophysics, University of Madras, Guindy Campus, Chennai, Tamil Nadu 600025 India

**Keywords:** Nipah virus, DFT, Molecular docking, Inhibitor, ADMET properties, Quantum descriptors

## Abstract

Favipiravir is found to show excellent in-vitro inhibition activity against Nipah virus. To explore the structure–property relationship of Favipiravir, in silico designing of a series of piperazine substituted Favipiravir derivatives are attempted and computational screening has been done to evaluate its bimolecular interactions with Nipah virus. The geometrical features of all the molecules have been addressed from Density Functional Theory calculations. Chemical reactivity descriptor analysis was carried out to understand various reactivity parameters. The drug-likeness properties were estimated by a detailed ADMET study. The binding ability and the mode of binding of these derivatives into the Nipah virus are obtained from molecular docking studies. Our calculations show greater binding ability for the designed inhibitors compared to that of the experimentally reported molecule. Overall, the present work proves to offers new insights and guidelines for synthetic chemists to develop new drugs using piperazine substituted Favipiravir in the treatment of Nipah virus.

**Supplementary Information:**

The online version contains supplementary material available at 10.1007/s42452-020-04051-9.

## Introduction

Nipah virus (NiV) has been identified as a zoonotic virus belonging to the genus Henipavirus, within the subfamily Paramyxovirinae [1, 2]. NiV is transmitted to humans through various direct sources such as animals and contaminated food and even through another infected person [3]. The virus was named after the village “Sungai Nipah” in Malaysia, where its first outbreak occurred in 1999 among pig farmers [4]. Then, another outbreak was reported in Bangladesh in 2001, and from then onwards outbreaks were reported annually in the country. Between 1998 and 2018, more than 600 cases of infection were reported, and the fatality rate was estimated to be 40–75% [5]. In India and Bangladesh about 75% and 51% of cases respectively were through human transmission, especially among hospital staff or visitors in the health care settings [3]. The source of infection in most cases was due to the consumption of raw date palm sap contaminated with saliva or droppings of the infected fruit bat owing to the fact that the natural reservoir of the virus is the fruit bats of the genus Pteropus [6, 7]. Some of the common symptoms of NiV are respiratory diseases, fever, muscle pain, inflammation of the brain, and acute or late onset encephalitis, and in most cases, the incubation period is reported to be 5 days to 2 weeks [5]. In May 2018, another outbreak occurred in the state of Kerala, India, and of the 13 individuals infected, 11 died [8]. This situation urges us to come up with suitable remedial measures to eradicate NiV, which requires a complete understanding of NiV at the molecular level.

**Scheme 1:**
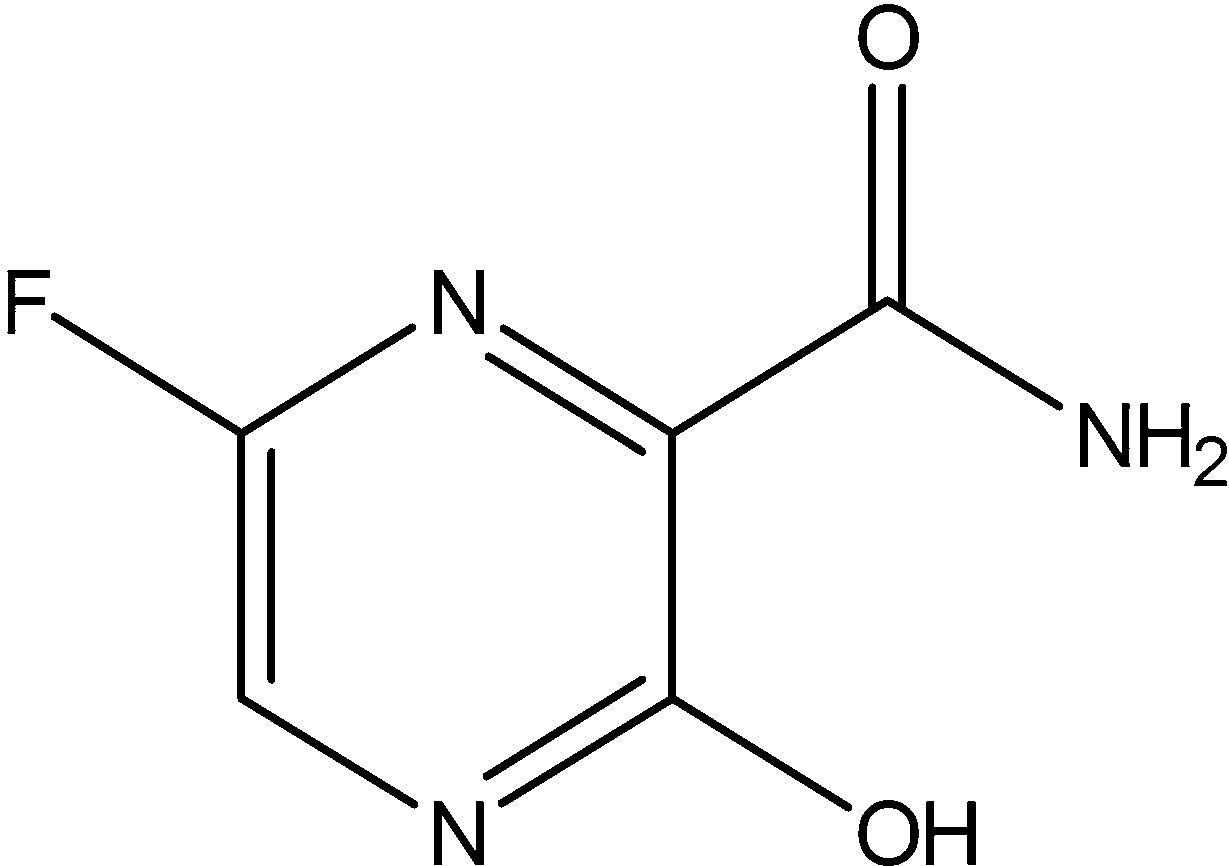
Molecular structure of Favipiravir

The structure of NiV with glycoprotein (G) attachment has 602 amino acid residues where the glycoprotein functions as a receptor-binding protein providing attachment to host cell receptors. This protein plays a vital role in facilitating the fusion of cell membrane with the virus through F protein by interacting with Ephirin-B2 receptors present on host cell and thereby making it an effective target for inhibition [9–12]. The World Health Organization (WHO) has set NiV as a priority disease on the WHO R&D Blueprint [3]. In 2006 Georges-Courbot et al. [13, 14] has identified that ribavirin drug to delay death by 2 days but unfortunately could not prevent death. Later in 2009, although a combination of antimalarial drugs, chloroquine, and ribavirin was used to treat NiV, it caused side effects [15]. A milestone in NiV research was achieved in 2018 when Dawes et al. [16] revealed the antiviral effect of favipiravir against live NiV isolates. Favipiravir (6-fluoro-3-hydroxy-pyrazine-2-carboxylic acid amide), shown in Scheme 1, has demonstrated a 100% survival rate against NiV and had already been licensed in Japan for use against influenza viruses [17–19]. Invitro studies have shown broad spectrum anti-RNA virus activities of Favipiravir with life threatening viruses such as Ebola virus, rhinovirus, and Lassa Virus [20]. However, studies on the structural modifications of favipiravir to enhance its activity against diseases are yet to be explored. Though intensive support care has been recommended to treat severe respiratory problems and neurological complications associated with NiV [21], currently, no antiviral drugs or vaccines are available for NiV-infected individuals, and most importantly, no therapeutic strategy has yet been established against NiV.

In recent years, researchers have shown keen interest in investigating the relationship between the chemical structure of a drug and its physicochemical properties, which in turn can regulate the pharmacological activity of the drug molecule [22]. Taylor et al. [23] evaluated the ring structures in drugs listed in the FDA Orange Book and reported the 100 most frequent ring systems. According to their findings, benzene ring was the frequently reported ring system, followed closely by pyridine and piperidine. Piperazine, cyclohexane, tetrahydropyran, imidazole, and pyrrolidine were the other frequently occurring ring structures in drug molecules. In literature, piperazine ring is considered to be an important structural motif in drug discovery due to their wide biological applications [24–26]. Pyrazine ring–containing drugs are widely used as diuretics, anti-inflammatory agents, antidepressants, and anti-infectives (bactericides and fungicides) in the field of medicine [27]. Recent works on the synthesis of pyrazine-2-carboxylic acid derivatives of piperazine using commercially available, inexpensive T3P coupling suggest it to be a promising active component in biomedical applications [28]. A recent in silico study on bioisosteres of favipiravir obtained by substituting the fluorine atom with other halogens and closed rings was attempted against NiV [10]. Inspired by the experimental works on pyrazine-2-carboxylic acid derivatives, the present investigation deals with the designing of N-substituted piperazine derivatives of favipiravir and their activity against NiV. With all this prior knowledge, a density functional theory (DFT) study was taken up to look into the geometric features, electronic property, and ADMET properties have been carried out. Further, molecular docking analysis has been carried out to bring out the binding ability of these favipiravir derivatives for the inhibition of NiV.

## Materials and methods

### Density functional theory analysis

Density functional theory calculations were used for the optimization of the molecules chosen for this study at the B3LYP/6-311++*g*(*d*,*p*) level using the G09 program [29]. To confirm their ground state stability, frequency calculations were performed, and no imaginary frequency was obtained for any of the molecules. To obtain an in-depth understanding of the chemical reactivity and stability of these molecules, frontier molecular orbital (FMO) and electrostatic potential (ESP) analyses were performed.

### ADMET and drug-likeness prediction

The drug likeness and ADME (Adsorption, Distribution, Metabolism, Excretion) properties of these molecules were evaluated by SWISSADME server [30]. Toxicity studies were performed by ProTox-II online tool [31]. Pharmacological properties and pharmacokinetics of these molecules, that is, solubility (ESOL), gastrointestinal (GI) absorption, blood–brain barrier penetration and Lipinski’s rule of violations were analyzed.

### Molecular docking and binding energy estimation

Molecular docking was carried out by using Maestro [32]. GLIDE—6.6 searches were performed to understand the mode of binding and affinity of favipiravir derivative structures against NiV glycoprotein (PDB ID: 3D11). Three-dimensional structure of target NiV glycoprotein was used for the protein preparation wizard of Schrodinger. No hydrogen atoms were minimized until the average root mean square deviation reached a default value of 0.3 A. Sitemap 2.3 The prepared proteins were loaded on the workstation and the grid values were calculated about 45 Å to cover the entire protein amino acids for finding the binding conformation. Ligprep 2.3 modules (Schrodinger, 2014-2) were used for optimized compounds ligand preparation [33]. All molecular modelling studies were applied using OPLSAA (Optimized Potential Liquid Simulation for All-Atom) force field [34]. About 20 conformational poses were created and analysed for the best conformation poses. The docked poses were visualized using Maestro and PyMol [35].

## Results and discussion

**Scheme 2:**
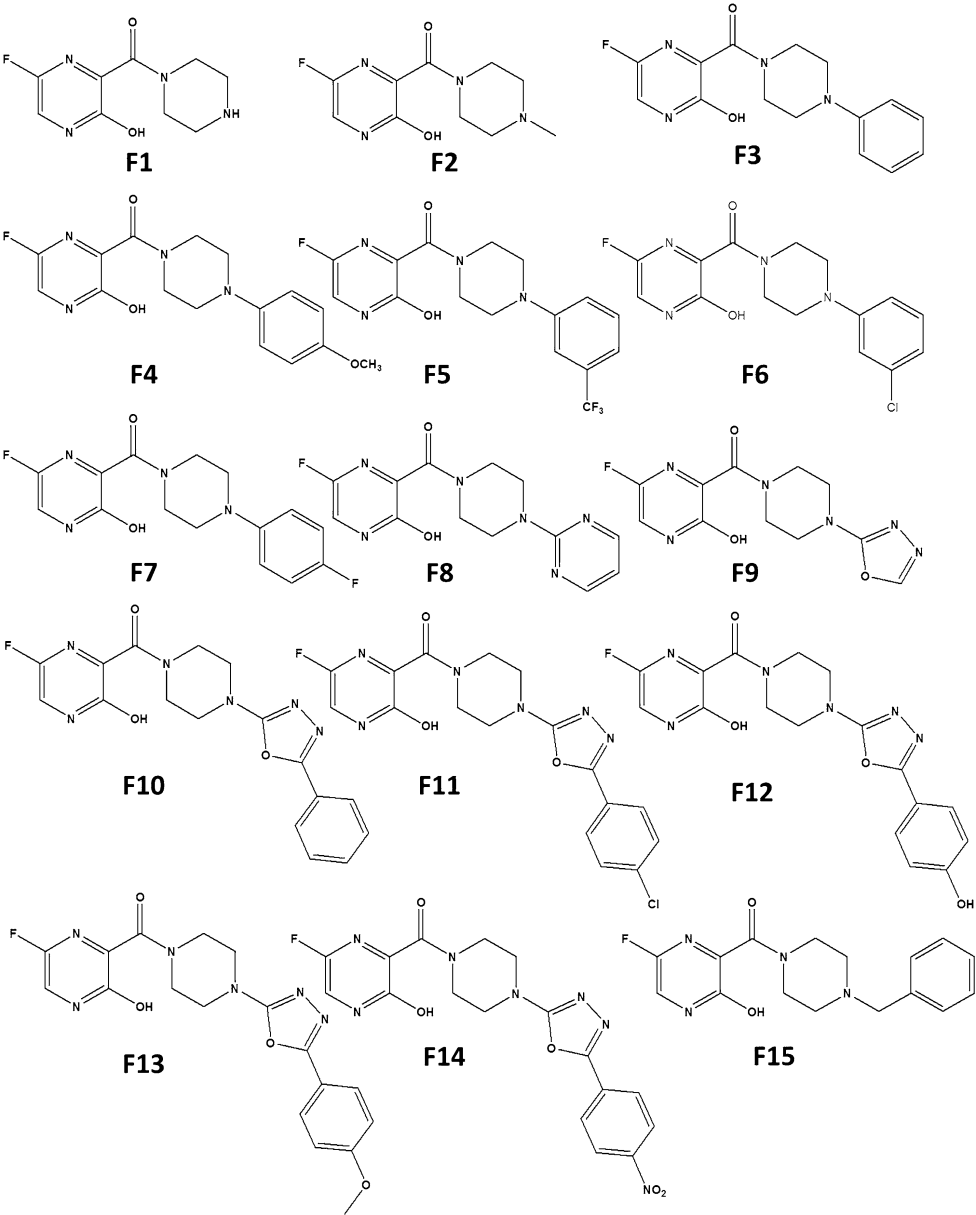
Molecular structure of the studied piperazine substituted Favipiravir derivatives

The favipiravir derivatives considered for this present work is shown in Scheme 2. Throughout this study, the acronym F refers to the favipiravir derivative, and the molecules are designated as F1, F2, F3,.., F15. The molecules F1 and F2 are alkyl-substituted piperazine derivatives of favipiravir. Similarly, F3 to F7 are phenyl-substituted derivatives, whereas in F8 a pyrimidine group is attached in place of phenyl moiety. Since the 1,3,4-oxadiazole group exerts antiviral, antimalarial, anticancer, and anti-inflammatory effects on compounds, F9-F14 are designed with oxadiazole substituents. The molecule F15 is a benzyl-substituted derivative of favipiravir.

### Structural analysis

**Fig. 1:**
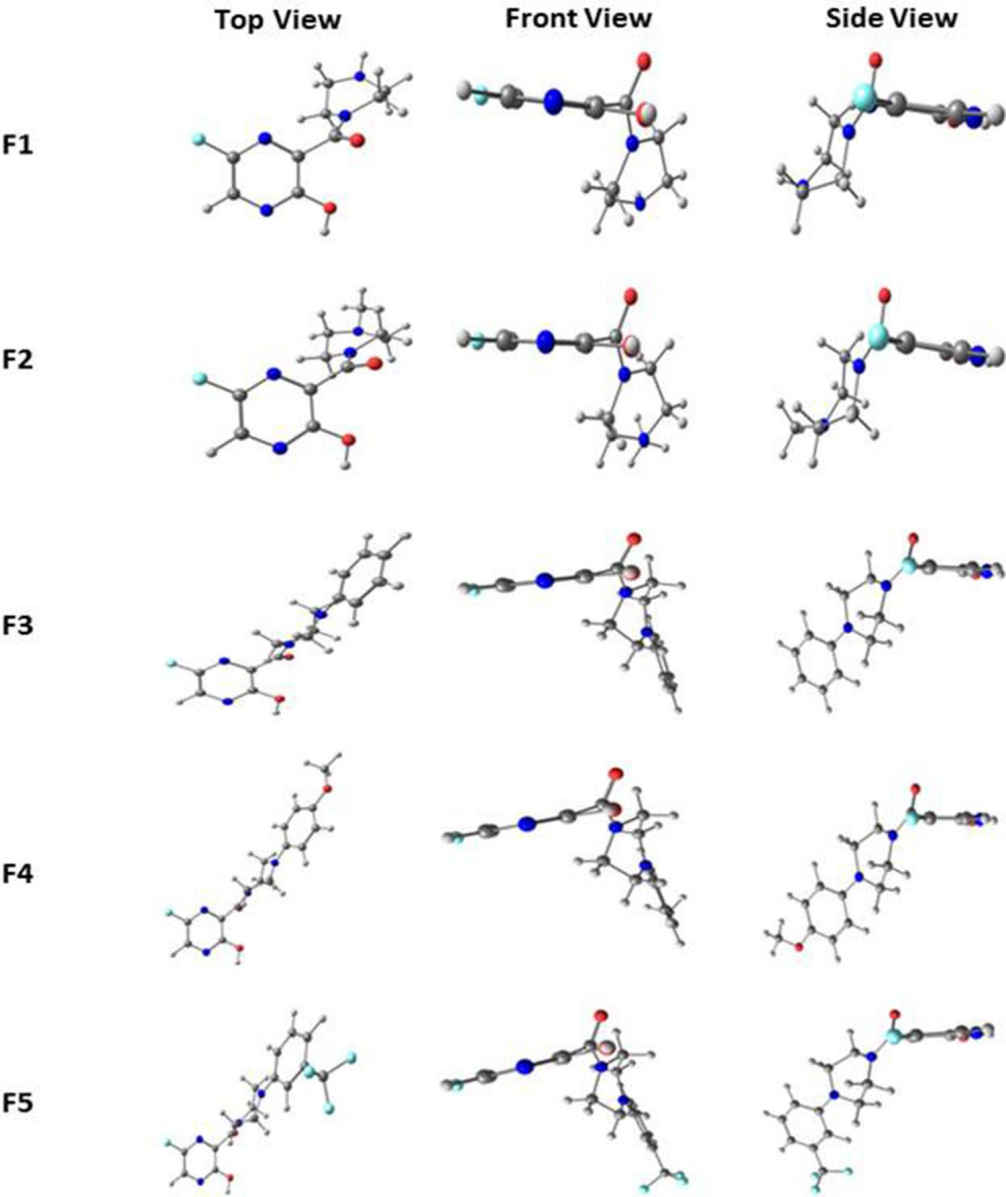
Optimized structures of Favipiravir derivatives 1 to 5 at B3LYP/6-311+ +*g*(*d*,*p*) level

**Fig. 2:**
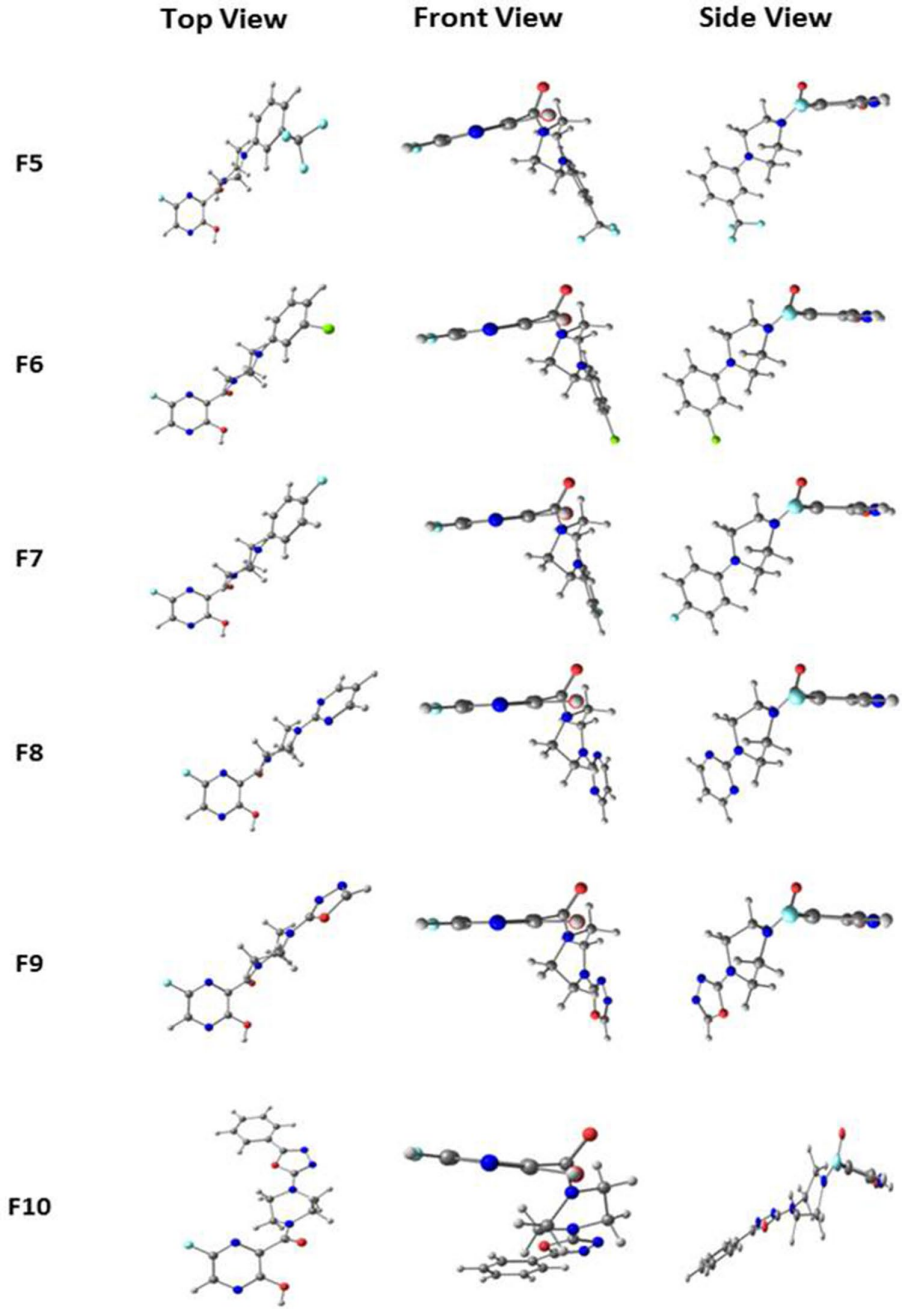
Optimized structures of Favipiravir derivatives 6 to 10 at B3LYP/6-311++*g*(*d*,*p*) level

**Fig. 3:**
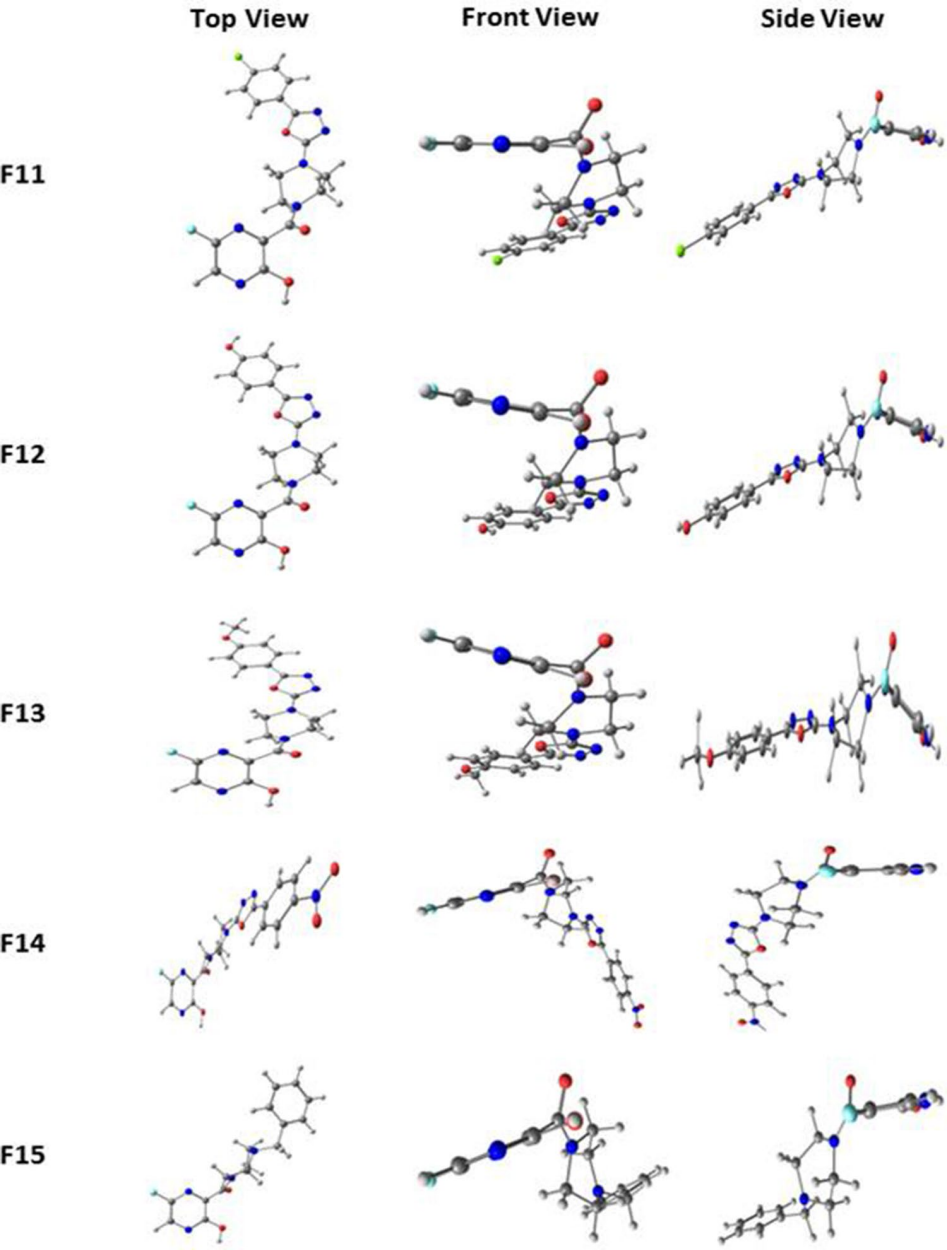
Optimized structures of Favipiravir derivatives 11 to 15 at B3LYP/6-311++*g*(*d*,*p*) level

The favipiravir derivatives (F1–F15) were optimized at the B3LYP/6-311++*g*(*d*,*p*) level. Figures 1, 2 and 3 illustrate the optimized structures of different derivatives of favipiravir substituted at the piperazine entity, and the selected bond parameters are presented in Table 1. The 6-fluoro-pyrazine unit is considered to be the parent unit of favipiravir derivatives throughout. When -NH_2_ group on the parent pyrazine entity is substituted with a piperazine ring, the structure distorts to become non-planar. The front- and side-view structures indicate the non-planar nature of these molecules. The dihedral angle between the parent unit and the piperazine ring lies between 61° and 79°, indicating the extent of distortion from planarity upon substitution. The bond length between C4 and C8 remains unchanged in all molecules, as shown in Table 1. It is noted that different substitutions at N23 (in piperazine ring) retain the non-planarity caused by the piperazine ring. The bond distance between the nitrogen in piperazine ring (N23) and the adjacent atom (R) attaching the substituent with the N23 was studied. The nature of N23-R bond varies with different R and the group associated with it. In molecules F9–F14, the substitution of the oxadiazole group at N23 makes the N23-R bond partially double bonded. This is due to the participation of lone pairs from N23 in resonance with the substituted unsaturated rings. Partial double bonds are also seen when pyrimidine is substituted in place of oxadiazole (F8). In the case of phenyl substitutions (F3–F7), the bond remained single as the phenyl group did not involve the lone pairs of N23 in resonance, making the bond weaker compared to others. In F15, it can be noticed that attaching a *sp*^3^-hybridised unit further weakens the N23-R bond. The bond between the carbonyl group and N10 is found to be partially double bonded in all cases. This can be attributed to the shuttle of lone pair of electrons between N10 and the carbonyl group. The intramolecular hydrogen bonding interaction between H12 and N6 was noted for all molecules, and it has been observed that the hydrogen bonding interactions remain constant for all molecules. The observed O–H…N6 hydrogen bond distance between H12 and N6 is 2.28 Ǻ, which lies well within the strong hydrogen bond limits according to Jeffrey [36]. From the optimized geometries, it is clear that piperazine unit is in plane with all its substituents except with the benzyl group. In the case of F15, the substituted group and the parent pyrazine group are in the same axis linked by a piperazine ring at right angle to the two units. Literature shows that dipole moment of a molecule renders useful information about the polarity of a given molecule [37]. It should be noted that due to dipole–dipole interaction the molecule that has a high dipole moment must have a greater tendency to interact with other molecules. This relationship between the dipole moment of molecules and interactions with other molecules is not comprehendible at all times; in some cases, a decrease in dipole moment may also cause an increase in interactions [38]. The dipole moment of the computed molecules shows a high variation among them, and therefore, no generalised conclusions could be drawn. Table 1 represents the dipole moment and dihedral angles of all molecules studied.

**Table 1:** Selected bond lengths (Å) and dihedral angles (°) and dipole moment calculated for favipiravir derivatives (1–15) at B3LYP/6–311+ +G(*d*,*p*) level

	Bond distances			Dihedral angle	Dipole moment
	C4–C8	N23-R	OH–N^a^		
F1	1.516	1.015	2.290	77.439	2.997
F2	1.517	1.455	2.290	78.868	3.586
F3	1.514	1.389	2.842	63.480	2.799
F4	1.515	1.365	2.284	64.200	3.573
F5	1.514	1.349	2.285	62.860	5.911
F6	1.515	1.350	2.289	69.280	5.492
F7	1.515	1.490	2.289	69.120	5.738
F8	1.515	1.351	2.288	69.330	4.668
F9	1.515	1.351	2.289	70.030	4.892
F10	1.513	1.345	2.285	61.240	6.331
F11	1.515	1.396	2.284	64.200	2.038
F12	1.514	1.384	2.285	62.750	1.857
F13	1.514	1.385	2.285	63.230	1.537
F14	1.514	1.390	2.284	62.820	3.155
F15	1.515	1.461	2.284	65.420	3.543

^a^Bond distance caused intermolecular hydrogen bonding within the pyrazine molecule

### Frontier molecular orbital analysis

**Fig. 4:**
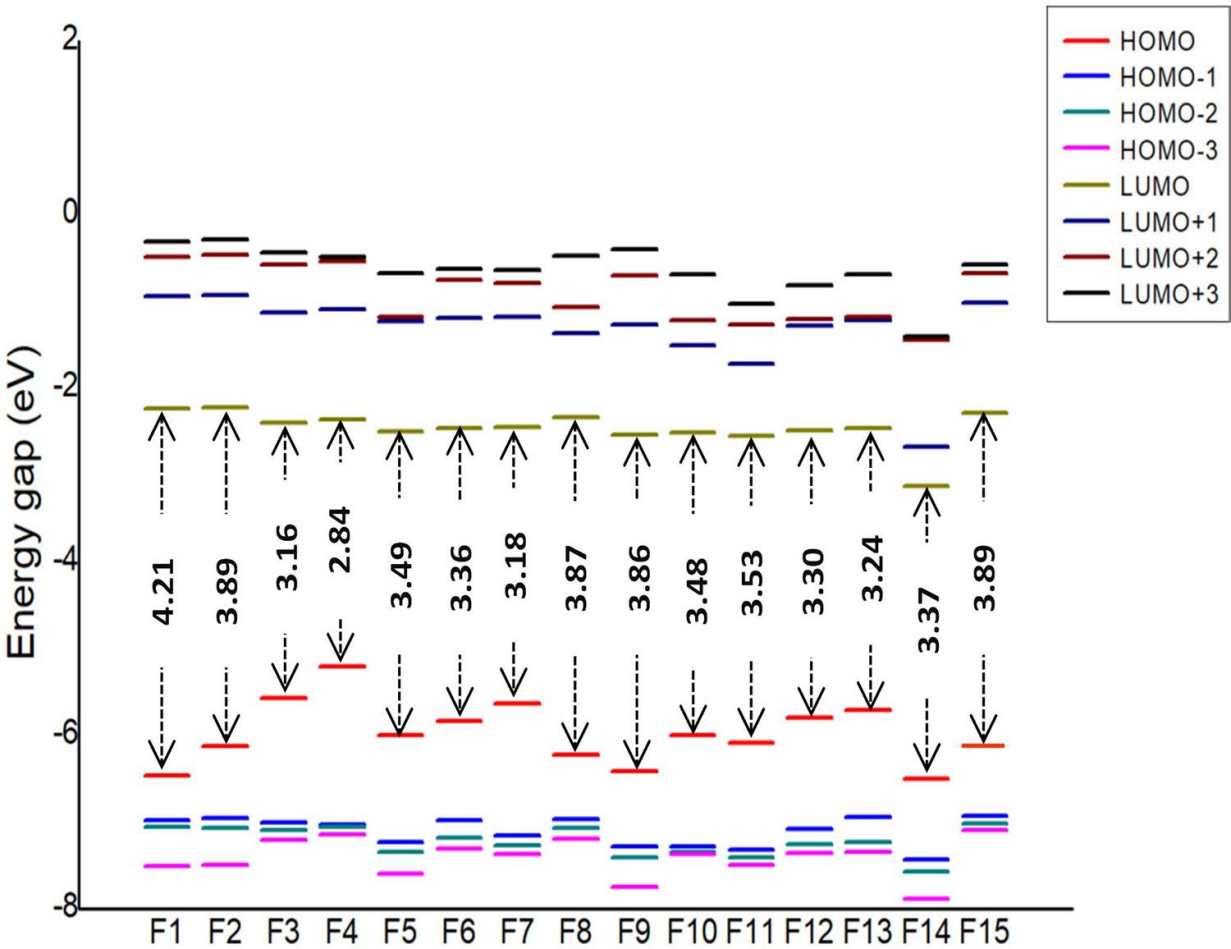
HOMO–LUMO energy gap (eV) of Favipiravir derivatives

**Fig. 5:**
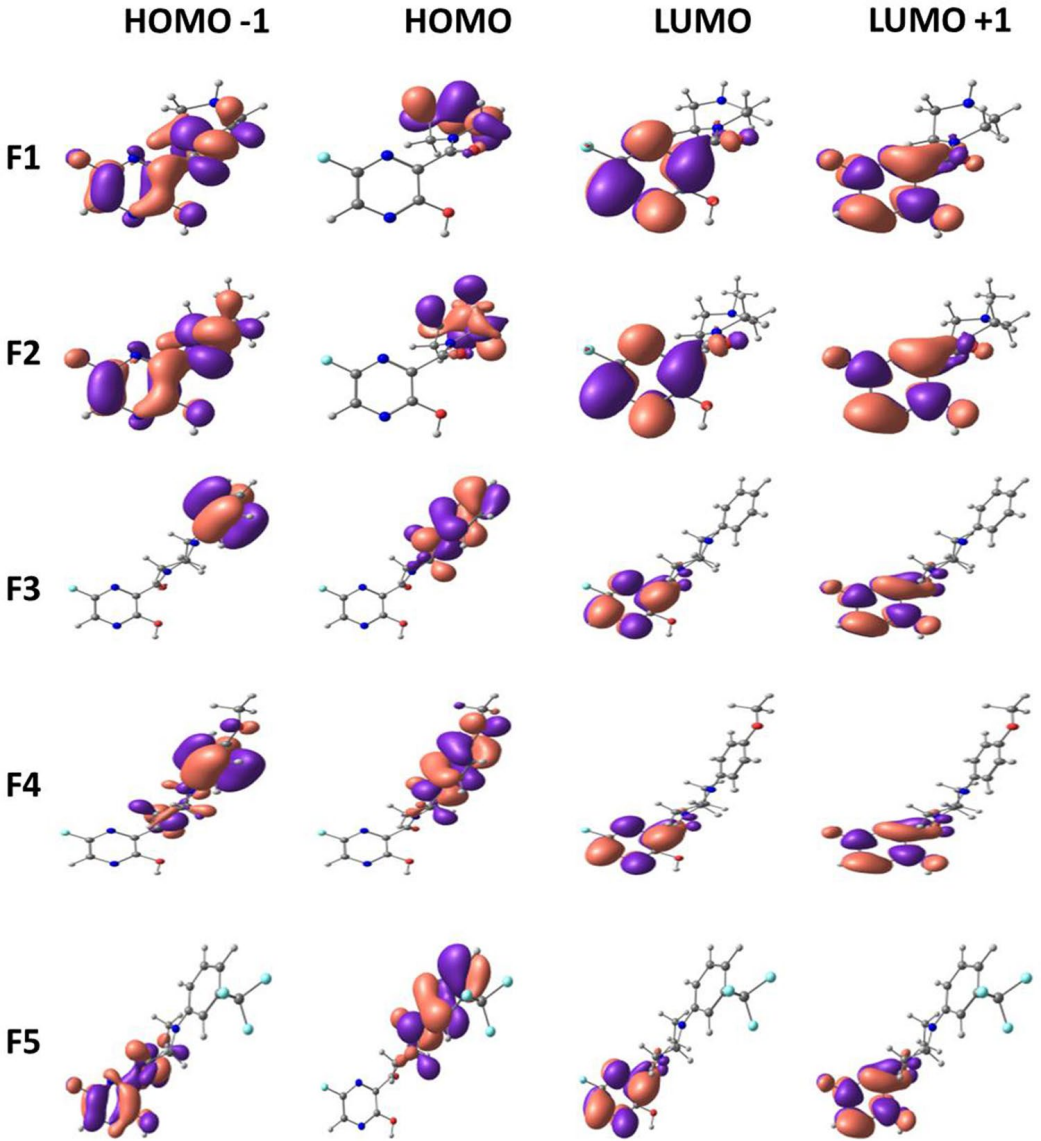
Frontier molecular orbitals of Favipiravir derivatives 1–5

**Fig. 6:**
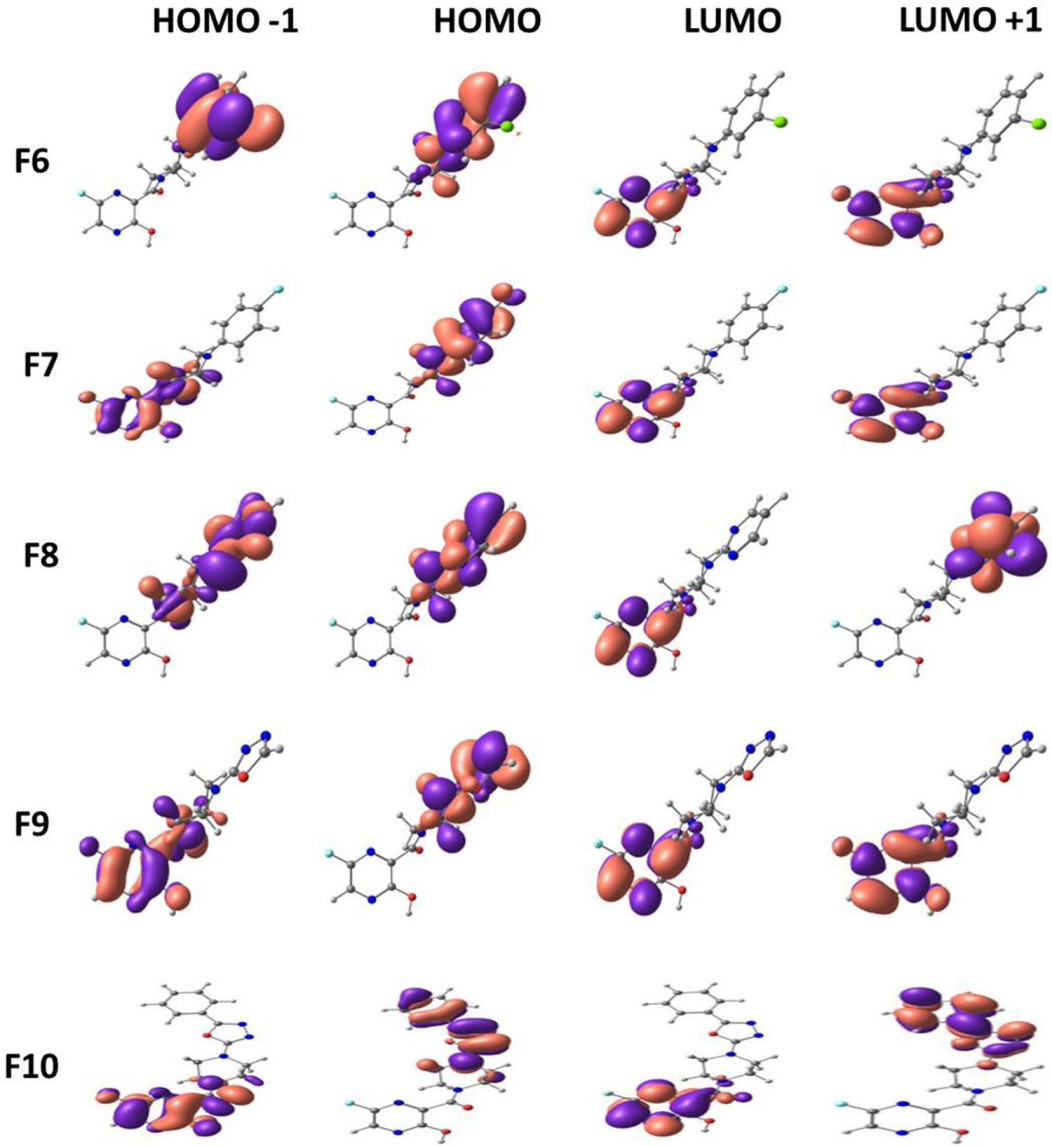
Frontier molecular orbitals of Favipiravir derivatives 6–10

**Fig. 7:**
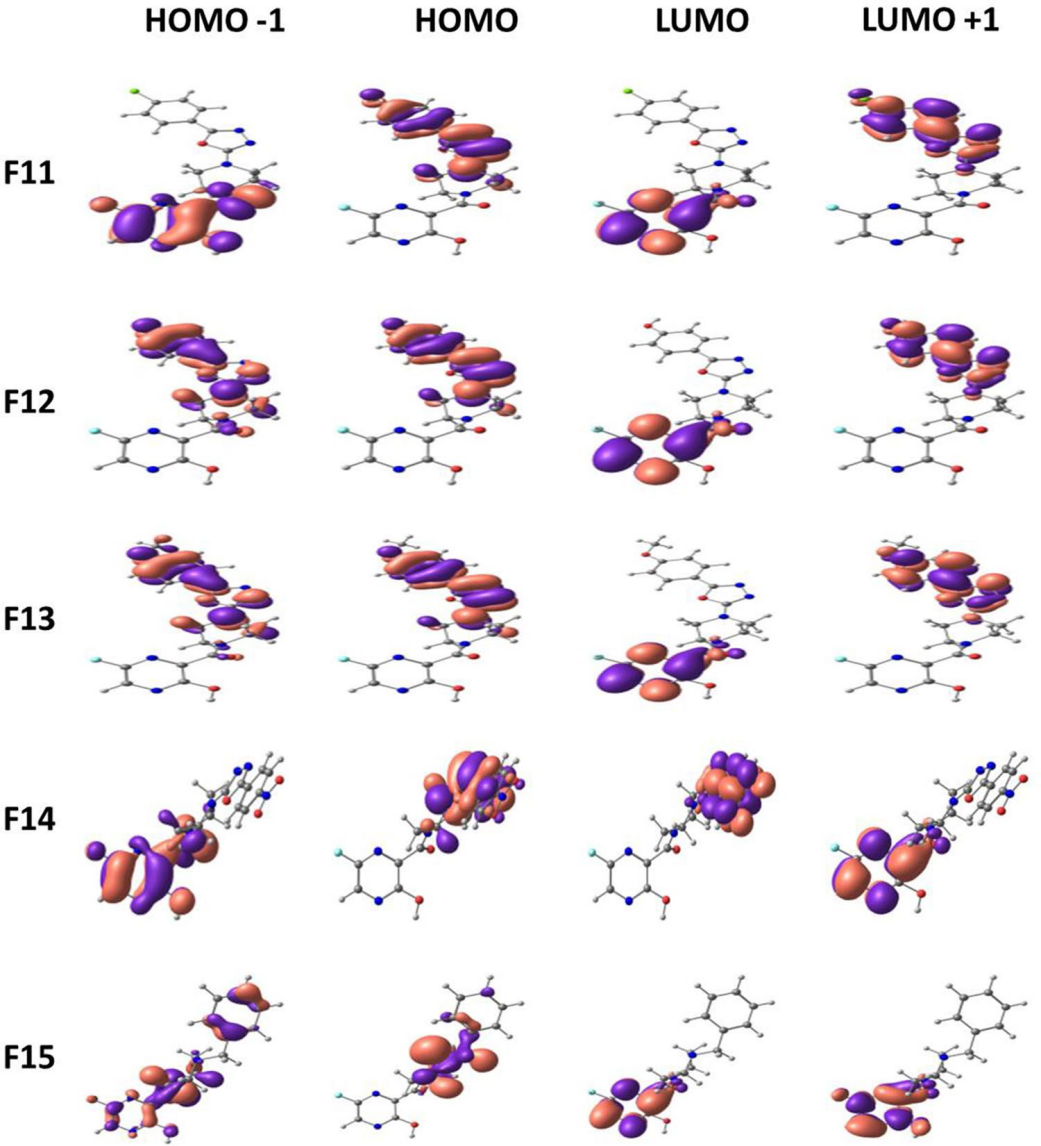
Frontier molecular orbitals of Favipiravir derivatives 11–15

Frontier molecular orbitals of molecules are of great importance in determining the chemical reactivity of molecules [39, 40]. It is reported that the electron-donating and electron-accepting abilities of molecules can be understood from FMOs. The energy gap or HOMO–LUMO (H–L) gap obtained from FMO analysis is a significant parameter that measures the intermolecular charge transfer and kinetic stability that indeed have been extensively used to explain biological activities [41, 42]. In general, a large energy gap is associated with low chemical reactivity and high stability, whereas a smaller gap represents more reactivity and less stability [43, 44]. The FMO energies are depicted in Fig. 4 with their corresponding energy gap. Among the 15 molecules, F4 shows the least HOMO–LUMO gap with 2.84 eV, and the highest is shown by F1with 4.21 eV. The H–L gap increases in the following order: F4 < F3 < F7 < F13 < F12 < F6 < F14 < F10 < F5 < F11 < F9 < F8 < F2 < F15 < F1. Upon the introduction of the phenyl group at the piperazine ring, the HOMO–LUMO gap further reduces up to a minimum of 2.8 eV. Figures 5, 6 and 7 illustrate the FMOs of all 15 molecules visualized using Chemcraft 1.8 software [45]. The HOMOs of all the designed molecules are found to be localized on the substituted group and the piperazine ring than over the pyrazine ring. An exception to this trend is noticed in F15, where the HOMO is occupied on the piperazine and is not delocalized over the attached benzyl group. This is due to the non-planar nature observed between piperazine and benzyl group. The LUMOs lies on the parent pyrazine unit except in F14. The derivative F14 has its LUMO on the substituted phenyl group due to the electron-withdrawing effect of -NO_2_ group attached to the phenyl ring. Replacing the phenyl group with oxadiazole reduces the gap to 3.24 eV (F13). Pyrimidine ring, on the other hand, increases the H–L energy gap to 3.16 eV.

**Scheme 3:**
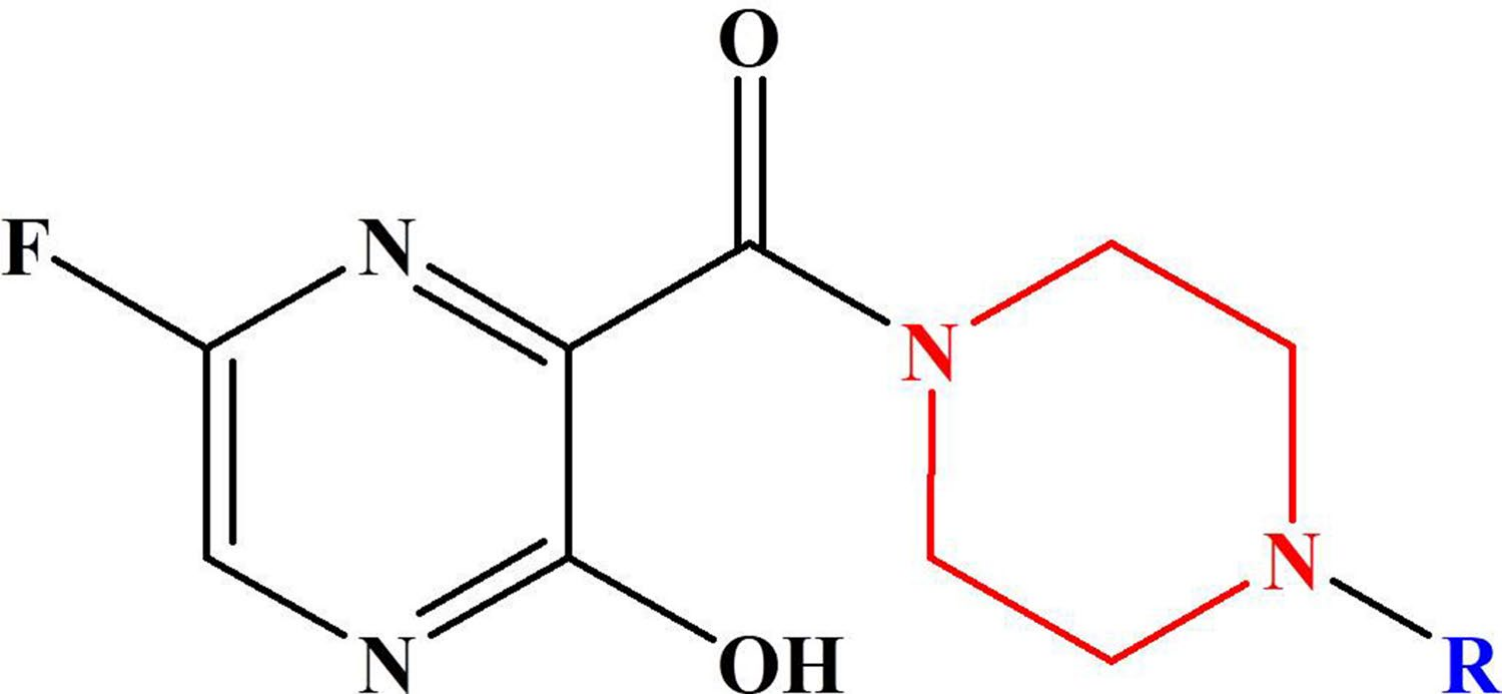
General representation of the different regions of Favipiravir derivatives used for % contribution analysis

**Fig. 8:**
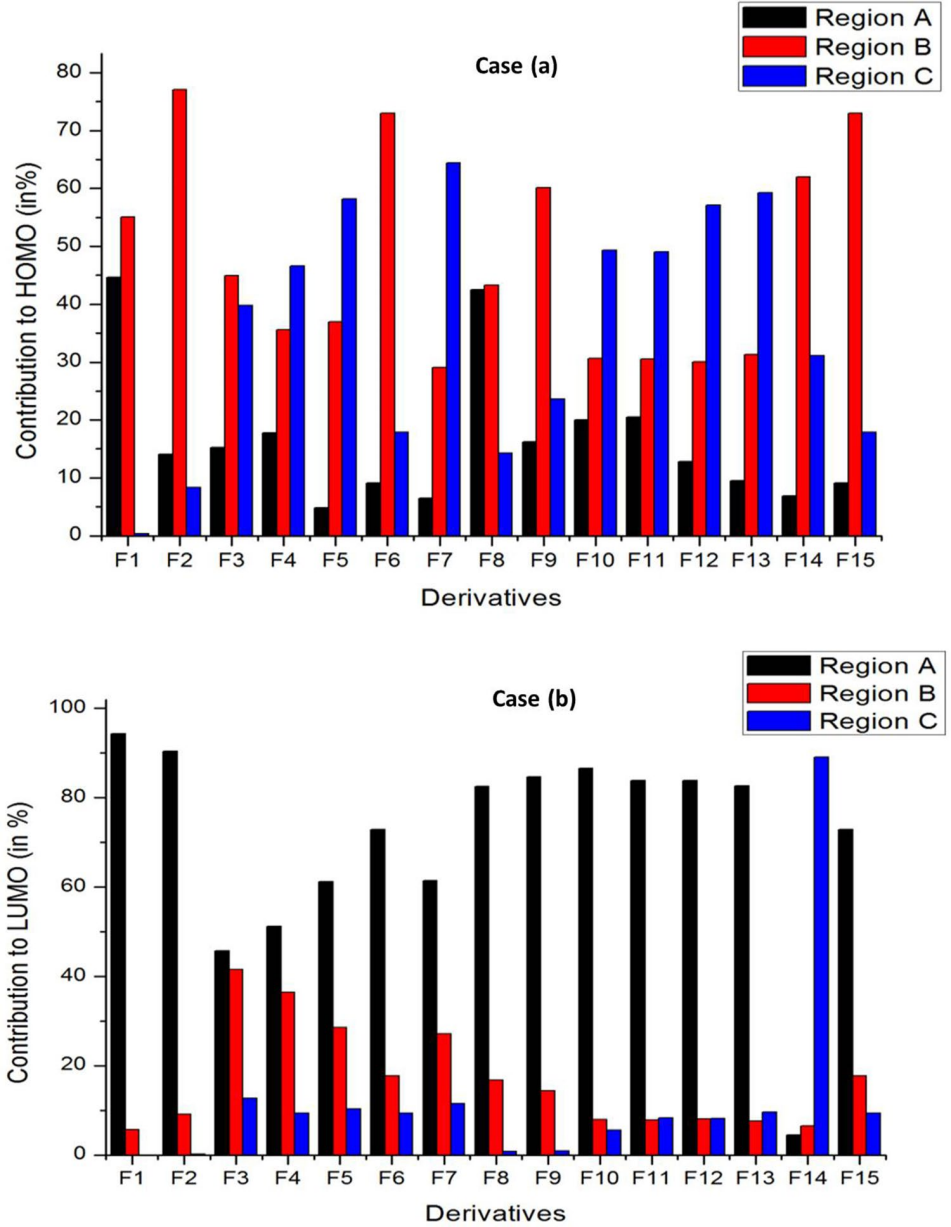
Percentage contribution of different groups of Favipiravir derivatives towards frontier molecular orbital (FMO) analysis. Case **a** depicts contribution to HOMO and Case **b** contribution to LUMO

To envisage the FMOs, the energetics of the FMOs were analysed using QM Forge software [46]. This analysis helps in understanding the contributions of different moieties of the molecules towards their HOMOs and LUMOs. Here, as shown in Scheme 3, the molecule is segmented into three parts, namely, region A consisting of the pyrazine ring, region B consisting of the piperazine ring, and region C involving the different substitutions on the piperazine ring. Figure 8 depicts the contribution from each part to HOMO (Fig. 8a) and LUMO (Fig. 8b). Figure 8a shows that the pyrazine ring contributed maximum (~ 80%) to the stabilisation of HOMO followed by region C consisting of different substituents. In molecules with the pyrimidine group as the substituent, region A and region B contribute equally to the HOMO contradicting all other substitutions. For instance, molecule F4 got 42.5% from region A and 43.3% from region B. Because there are piperazine ring substitutions in F1 and F2, the contribution from region C is very low and therefore negligible. Figure 8b shows that the major contribution of LUMO is from region A in all molecules except in F14. The contribution from region C is 89%, which is distinct from other substituents with 0–10% contribution. These analyses of FMOs help in synthesizing molecules with desired HOMO and LUMO levels.

### Global descriptors

The molecular properties that govern the reactivity and selectivity of the compounds are estimated using the Koopman’s theorem relating the energy of HOMO and LUMO as reported [47, 48]. The energy of HOMO is related to ionization potential (*I*), whereas the energy of LUMO is related to the electron affinity (*A*) of the molecule. The global reactivity descriptors such as chemical potential (*μ*), electronegativity (*χ*), hardness (*η*), softness(*S*), and electrophilicity index (*ω*) are calculated using the HOMO–LUMO energies [49–51]. These quantum chemical descriptors are calculated using the following equations:

1 $${\text{Ionization potential }}\left( I \right) = - E\left( {{\text{HOMO}}} \right)$$

2 $${\text{Electron}}\,{\text{affinity}}\left( A \right) = - E\left( {{\text{LUMO}}} \right)$$

3 $${\text{Chemical}}\,{\text{potential}}\left( \mu \right) = - \left( {I + A} \right)/2$$

4 $${\text{Electronegativity}}\left( \chi \right) = \left( {I + A} \right)/2$$

5 $${\text{Hardness}}\left( \eta \right) = \left( {I - A} \right)/2$$

6 $${\text{Softness}}\,\left( S \right) = 1/2\eta$$

7 $${\text{Electrophilicity}}\,{\text{index}}\,\left( \omega \right) = \mu ^{2} /2\eta$$

It is well known that the higher the ionization potential, the higher is the energy required to remove an electron from the HOMO. A low value of electron affinity indicates the ease with which electrons can be removed from a molecule. A molecule with high electronegativity strongly attracts electrons from donor moieties. Chemical potential (*μ*) is a measure of reactivity and stability of a molecule. It refers to the unwillingness of a molecule to decompose into its elements easily. A negative chemical potential designates a molecule to be more stable. The chemical hardness and softness of the molecule dictate the polarisability of the molecule. A higher hardness and a lower softness confirm the less-polarisable nature of the molecules. Table 2 shows the global parameter calculated for the molecules studied. It can be noticed that F4 shows the lowest H–L gap as already explained in the FMO analysis. The ionization potential of F4 is the lowest (5.2 eV), and therefore, the electrons can be knocked out of the HOMO of F4 easily compared to other molecules. The electron affinity of F4 is the lowest (2.36 eV) among the molecules, suggesting the possibility to undergo electrophilic reactions. Meanwhile, it should be noted that F4 shows a high chemical reactivity, as inferred from its low H–L gap. As evident from the calculated ionization potential, the electronegativity of the F4 is the least among all others. All molecules have a negative chemical potential, indicating that all are stable and do not decompose into their elements easily. Electrophilicity index is the ability of a molecule to accept electrons [50, 52]. High softness, electrophilicity, chemical potential, and low electronegativity denote the reactivity of these molecules. Due to the presence of –NO_2_ group on the oxadiazole ring, F14 has the highest electronegativity value. F4, being the only molecule with a pyrimidine moiety, has shown appreciable values for *I*, *A*, *χ*, *μ*, *η*, and *S*. This analysis provides useful inputs for tuning the chemical properties of molecules for desired applications in biology [53].

**Table 2:** HOMO LUMO energies and the global parameters computed for the derivatives

	HOMO	LUMO	H–L gap (eV)	*I* (eV)	*A* (eV)	*Χ*	*μ*	*η*	*S*	*ω*
F1	− 6.463	− 2.245	4.218	6.463	2.245	4.354	− 4.354	2.109	0.237	4.495
F2	− 6.123	− 2.230	3.893	6.123	2.230	4.176	− 4.176	1.946	0.257	4.480
F3	− 5.564	− 2.402	3.163	5.564	2.402	3.983	− 3.983	1.581	0.316	5.016
F4	− 5.208	− 2.363	2.845	5.208	2.363	3.785	− 3.785	1.423	0.351	5.036
F5	− 5.997	− 2.503	3.494	5.997	2.503	4.250	− 4.250	1.747	0.286	5.169
F6	− 5.835	− 2.466	3.368	5.835	2.466	4.150	− 4.150	1.684	0.297	5.114
F7	− 5.637	− 2.457	3.180	5.637	2.457	4.047	− 4.047	1.590	0.314	5.150
F8	− 6.220	− 2.346	3.875	6.220	2.346	4.283	− 4.283	1.937	0.258	4.735
F9	− 6.413	− 2.548	3.865	6.413	2.548	4.481	− 4.481	1.933	0.259	5.194
F10	− 5.996	− 2.513	3.483	5.996	2.513	4.254	− 4.254	1.742	0.287	5.196
F11	− 6.087	− 2.554	3.533	6.087	2.554	4.320	− 4.320	1.766	0.283	5.283
F12	− 5.792	− 2.488	3.304	5.792	2.488	4.140	− 4.140	1.652	0.303	5.187
F13	− 5.714	− 2.469	3.245	5.714	2.469	4.092	− 4.092	1.623	0.308	5.159
F14	− 6.502	− 3.131	3.371	6.502	3.131	4.816	− 4.816	1.686	0.297	6.881
F15	− 6.185	− 2.285	3.899	6.185	2.285	4.235	− 4.235	1.950	0.256	4.600

### Molecular electrostatic potential

**Fig. 9:**
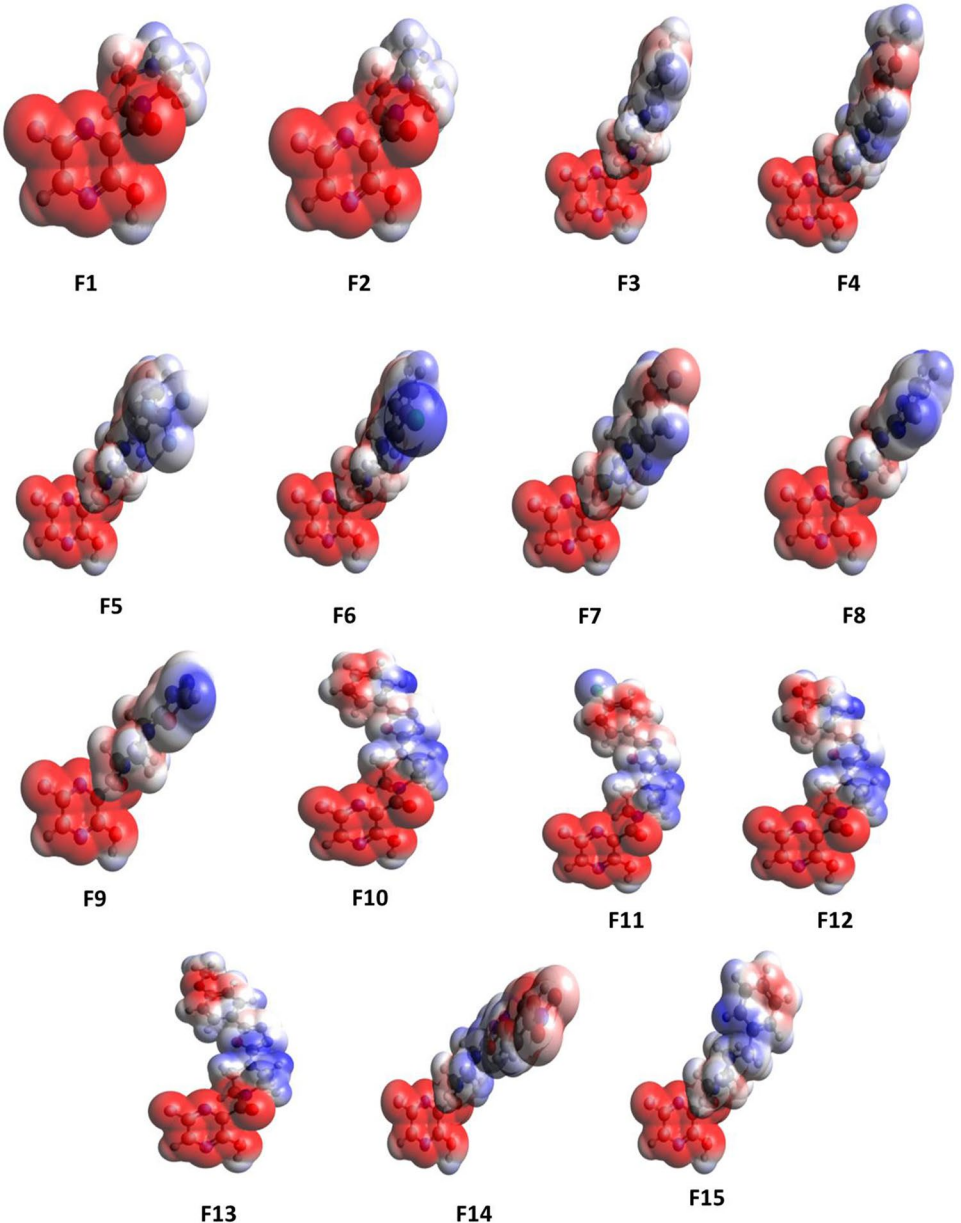
Molecular electrostatic positional (MEP) diagram of the derivatives (1–15)

Molecular electrostatic potential (MEP) surfaces are often used to evaluate the chemical reactivity, electronegativity, and dipole moment of the molecule [54–56]. MEP analysis is also used to understand the possible interactions of a molecule with its adjacent groups and surrounding. In addition, MEP is often used to identify the potential regions of molecules for possible hydrogen bonding, electrostatic and other stabilising interactions [57]. Therefore, MEP analysis was performed on all molecules using Avogadro software (Fig. 9) [58]. The distribution of ESP of the molecule is represented by red for the more electron-rich region with a negative potential and blue for the electron-poor regions with positive potential. It can be seen that the red region is mainly localized on the parent pyrazine moiety, indicating favourable sites for an electrophilic attack. The molecules that have strong electron-donating groups such as –OH,–OR, and -phenyl and weak electron-withdrawing groups (–Cl) in the substituents cause a patch of electron richness on the other end of the molecules. The presence of oxadiazole brings in electron density on it, making the centre of the molecule red in addition to the pyrazine ring. The positive ESP, that is, the electron-poor region, lies on the piperazine ring, indicating the nucleophilic sites on the molecule. The MEP is not symmetrically distributed over the molecule due to the non-planar nature. The white patches at random sites on the molecule indicate the absence of ESP.

### Pharmacological analysis

In silico ADMET prediction is considered as the first step in analysing new molecules for drug design in order to reduce the time wasted on lead compounds that would be toxic or metabolised to an inactive form [59–61]. Parameters such as water solubility, lipophilicity, and drug-likeness for the compounds are important for pharmacokinetics of the drug. Table 3 depicts some of the ADMET properties predicted by Swiss-ADME and ProTox II webserver tools. Solubility is an important property influencing the absorption of drugs meant for parental usage. A drug should be soluble in water to deliver an adequate quantity of active ingredients in the dosage [62, 63]. The studied molecules are soluble in water, which in turn facilitates absorption during oral administration. The lipophilicity of drugs described by the partition coefficient between *n*-octanol and water (log *P*_o/w_) is another significant factor affecting the pharmacokinetics of drug [64]. The log *P*_o/w_ values of molecules (Table 3) ranged between 1.16 and 2.41, indicating the solubility and permeability of the molecules. Molecular flexibility is calculated by the number of rotatable bonds is found to be a significant predictor of oral bioavailability [65]. The number of rotatable bonds of the studied molecules varies from 2 to 5, which indicates the flexibility of certain molecules than others. All molecules fulfil the Lipinski rule of five and are predicted to exhibit solubility in water, implying a good oral bioavailability [64, 66]. It is reported that bioavailability of drug is driven by GI absorption [67]. The GI absorption was studied, and the molecules had a high absorption, making it potential leads for drug discovery. In silico toxicity analysis is evolving as a platform for predicting the toxic effects of chemicals on humans, animals, and the environment [68–70]. Molecules were analysed for carcinogenicity, immunotoxicity, mutagenicity, and cytotoxicity, which are classified as toxicological endpoints in the Pro Tox II webserver. All molecules were identified as nontoxic based on different toxicities mentioned earlier (Table 3). The predicted median lethal dose (LD_50_) weight varies from 550 to 2000 mg/kg among all molecules and is thus categorized to be class 4 toxic.

**Table 3:** In silico ADMET prediction of designed compounds

	Log S	Lipinski	Log Po/w	# Rotable bonds	GI Absorption	pIC50	Toxicity			
							Carcinogenicity	Immunotoxicity	Mutagenicity	Cytotoxicity
F1	− 1.31	Yes	1.28	2	High	4.63	Inactive	Inactive	Inactive	Inactive
F2	− 1.68	Yes	1.39	2	High	4.64	Inactive	Inactive	Inactive	Inactive
F3	− 3.26	Yes	1.66	3	High	4.68	Inactive	Inactive	Inactive	Inactive
F4	− 3.33	Yes	2.12	4	High	4.69	Inactive	Inactive	Inactive	Inactive
F5	− 4.12	Yes	2.2	4	High	4.87	Inactive	Inactive	Inactive	Inactive
F6	− 3.42	Yes	1.75	3	High	4.65	Inactive	Inactive	Inactive	Inactive
F7	− 3.42	Yes	1.73	3	High	4.68	Inactive	Inactive	Inactive	Inactive
F8	− 3.32	Yes	1.64	4	High	4.38	Inactive	Inactive	Inactive	Inactive
F9	− 2.04	Yes	1.16	3	High	4.5	Inactive	Inactive	Inactive	Inactive
F10	− 3.57	Yes	2.2	4	High	4.86	Inactive	Inactive	Inactive	Inactive
F11	− 3.73	Yes	2.26	4	High	4.85	Inactive	Inactive	Inactive	Inactive
F12	− 3.43	Yes	1.82	4	High	4.86	Inactive	Inactive	Inactive	Inactive
F13	− 3.64	Yes	2.41	5	High	4.89	Inactive	Inactive	Inactive	Inactive
F14	− 3.63	Yes	1.83	5	High	4.8	Inactive	Inactive	Inactive	Inactive
F15	− 3.08	Yes	2.06	4	High	4.67	Inactive	Inactive	Inactive	Inactive
Favipiravir	− 1.13	Yes	0.70	1	High	4.34	Inactive	Inactive	Inactive	Inactive

### Predicted pIC_50_

The pIC_50_ values of all molecules are predicted using a web server developed by Manoj et al. [71] The pIC_50_ value of favipiravir was 4.66 in order to compare with its derivatives. The pIC_50_ values are tabulated in Table 3. The molecules with piperazine moiety showed high pIC_50_ values compared to favipiravir, except in the case of F8 (4.38). Substituted oxadiazole groups possessed high values among all molecules. Trifluoromethyl substitution in the phenyl group causes F5 to have higher pIC_50_ values as in the oxadiazole group. Understanding pIC_50_ values are of great importance in drug designing to provide useful information about the extent of inhibition possible.

### Biomolecular interaction studies

**Fig. 10.**
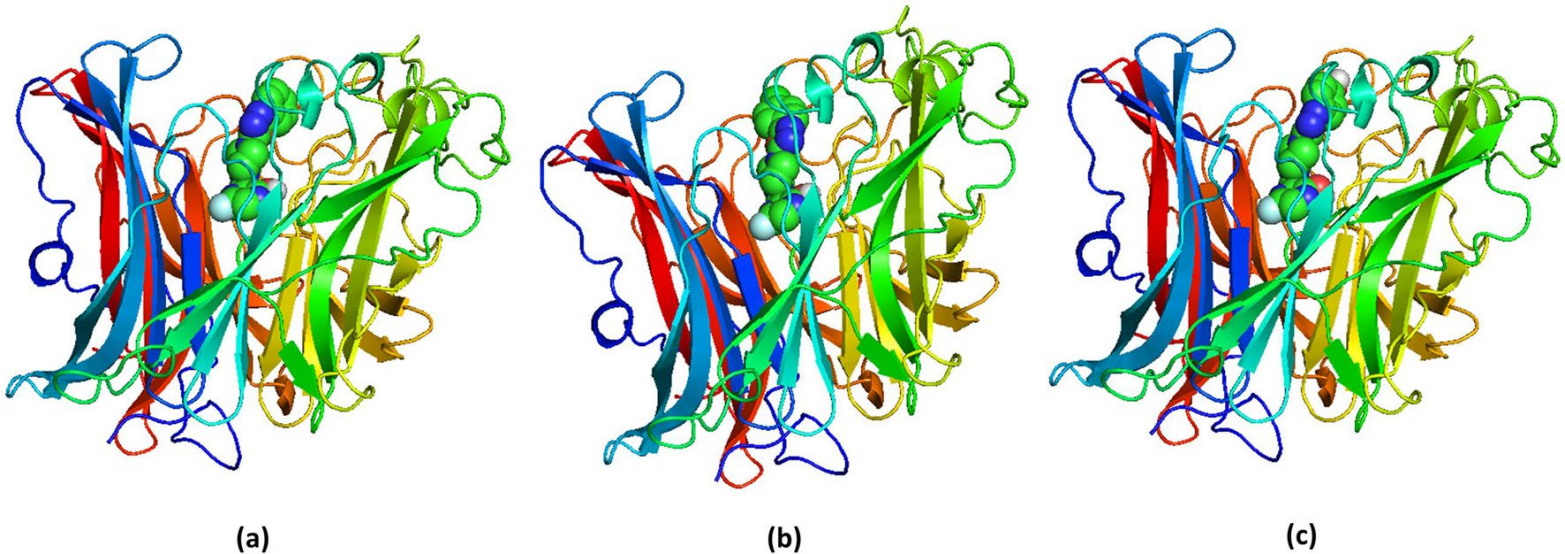
3D view of the binding conformations of molecule with highest binding energy at the active site of the protein for selected molecules **a** F10, **b** F11, **c** F12

**Fig. 11:**
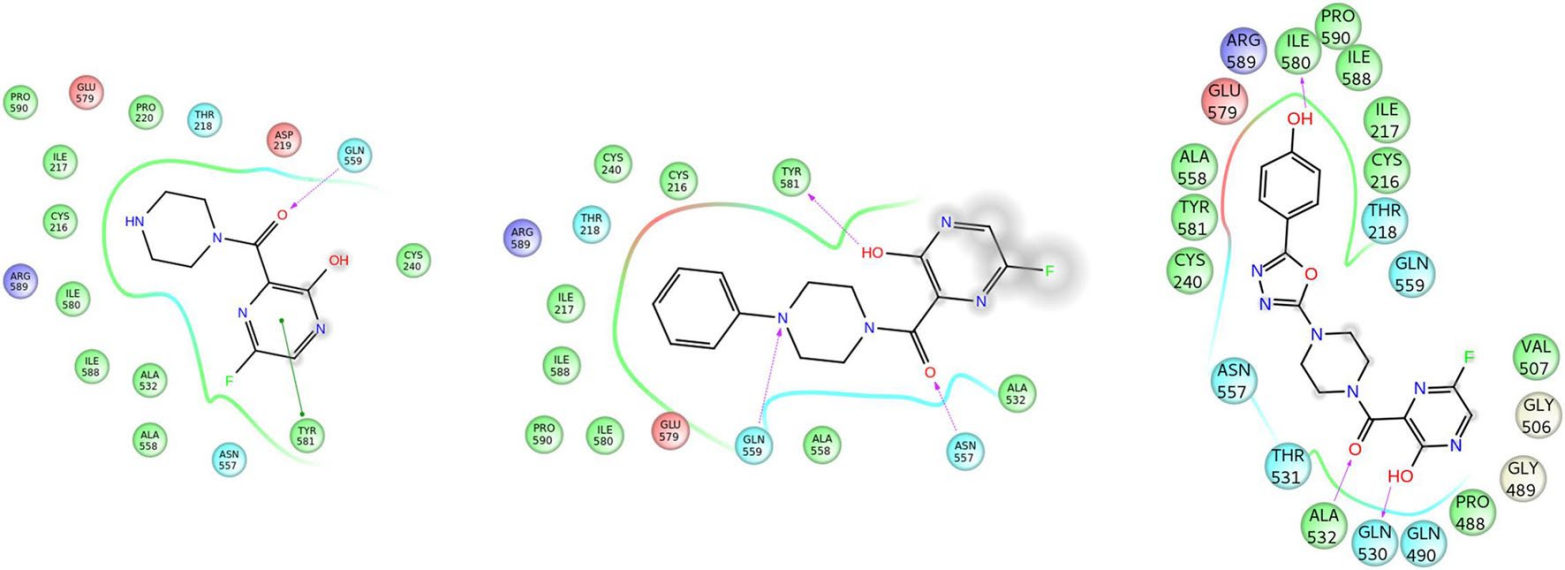
Schematic representation of interactions made by molecules with highest binding energy with the protein using Ligplot for selected molecules. **a** F10, **b** F11, **c** F12

Molecular docking is considered to be one of the most important methods in the discovery of small drugs [72–74]. In our study, molecular docking of all molecules was performed using Maestro glide docking program to understand the possible interactions between the protein and the molecule. The best-possible conformations obtained for the molecules (F1, F3 and F12) with the highest docking scores are shown in Fig. 10. The 2D view of the interactions produced by the molecules of high binding with the targeted protein are presented in Fig. 11, and the essential parameters are described in Table 4. The 3D view of the protein–ligand conformation and 2D view of the interactions of the remaining molecules are deposited in the supplementary material in Figures S1 and S2, respectively. As noted from the table, except F15 (− 4.961), F7 (− 5.222), F8 (− 5.506) and F9 (− 5.728) all other derivatives show significant binding with the protein. Oxadiazole-substituted molecule (F12) shows the highest value with -8.909. The binding pocket of F12 bound NiV protein includes the following residues: Gln490, Gln530, Ala532, Pro488, Thr531, Cys240, Tyr581, Ala558, Glu579, Arg589, Ile580, Thr218, Gln559. The BE of F15 is observed to be the lowest (−43.477). It is evident that understanding hydrogen bonding interactions along with other stabilizing interactions of molecules with the protein is vital in drug discovery. All molecules show hydrogen bonding interaction with the active-site amino acids of the protein. The number of H-bonding interactions between the amino acid and the molecule varies from 1 to 4. For instance, the molecules F3, F6, F11, F12, F13 and F14 show three hydrogen bonding interactions, whereas F1, F2 and F5 show only one hydrogen bonding. In this study, we can see that the number of H bonding interactions of a molecule cannot be correlated with BE. This is explained using F1 and F14, where the former with single H-bond shows the highest BE, and the latter though with three H-bonds is a poor binder.

**Table 4:** Molecular docking parameters of derivatives (1–15) against Nipah Virus (3D11)

	Docking score (kcal/mol)	Glide energy (kcal/mol)	H bonding residues	Hydrophobic interactions
F1	− 8.593	− 52.783	Gln559	Cys240, Tyr581, Ala558, Ala532, Ile588, Ile580. Cys216, Ile217. Pro590, Pro220
F2	− 6.894	− 49.383	Gln559	Ala532, Ile580, Ala558, Ile217, Pro590, Ile588, Cys216, Cys240, Tyr581
F3	− 7.939	− 54.604	Asn557. Gln559,Tyr581	Ala532, Ala558, Ile580, Pro590, Ile588, Ile217, Cys216, Cys240, Tyr581
F4	− 6.703	− 44.574	Thr531, Ala532, Asn557	Ala532, Ala558, Ile580, Cys216, Ile217, Pro590, Cys240, Ile588, Tyr581
F5	− 6.303	− 59.426	Tyr581	Ala532, Tyr581, Ala558, Ile580, Cys216, Ile588, Cys240, Ile217, Pro590, Pro220
F6	− 5.996	− 45.071	Gln559, Asn557, Tyr581	Ala532, Ala558, Cys240, Cys216, Ile217, Ile580, Ile588, Pro590, Try581
F7	− 5.222	− 40.967	Asn557, Ala532	Ala532, Ala558, Tyr581, Ile580, Ile588
F8	− 5.506	− 43.226	Gln559, Ile217	Cys240, Tyr581, Ile580, Ala558, Pro590, Ile588, Cys216, Ile217
F9	− 5.728	− 41.174	Gln559, Ile27	Ile580, Ala558, Tyr581, Cys216. Pro590, Cys240, Ile588, Ile217
F10	− 7.001	− 51.355	Gln559, Ile217	Ile580,Ala558, Tyr581, Cys240, Pro590, Ile217, Cys216, Ile588, Val587
F11	− 7.526	− 51.079	Ala532, Gln530, Gln559	Val507, Pro488, Ala532, Tyr581, Ala558, Pro590, Ile217, Ile588, Ile580, Cys216, Cys240
F12	− 8.909	− 55.229	Gln530, Ala532, Ile580	Val507, Pro488, Ala532, Cys240, Tyr581, Ala558, Ile580, Pro590, Ile588, Ile217, Cys216
F13	− 7.121	− 51.398	Gln530, Gly489, Gln559	Pro488, Val507, Leu526, Ala532, Tyr581, Ala558, Ile217, Ile588, Pro590, Ile580, Cys240
F14	− 5.655	− 49.305	Gln530, Ala532, Ile580	Ala532, Val507, Tyr581, Cys240, Ala558, Ile588, Ile580, Pro590, Ile217, Cys240, Tyr581
F15	− 4.961	− 43.477	Asn557, Gln559	Ala532, Ala558, Cys240, Cys216, Ile217,Ile588, Ile580, Tyr581, Pro590

The hydrophobic interactions are crucial in understanding the binding of molecules to the active site. Analysing the 2D figures suggests that the number of hydrophobic interactions exerted is related to the BE. For instance, F1, F11, F12 has more than 10 hydrophobic interactions, making it’s BE higher than the other derivatives. For the molecules with very low BE as in F7, the number of hydrophobic interactions is 5. This indicates that these molecules are stabilized inside the protein by hydrophobic interactions in addition to hydrogen bonds. In molecules F1, F4, F9, F10 and F14, the molecule is exhibiting pie stacking interactions with the protein Tyr581.It was noted that the number of rotatable bonds of the molecule analysed in the previous section influences the extent of binding to the protein. This attribute could be observed in the case of F10–F14 where the number of rotatable bonds range from 4 to 5, making them potential derivatives for NiV inhibition. These molecular docking stimulations would enable to understand the possible interactions of the molecule with the protein and help in designing potent inhibitors.

## Conclusion

In the present investigation, quantum mechanical calculation using DFT at B3LYP/6-311+ +*g*(*d*,*p*) method on 15 piperazine-substituted derivatives of favipiravir was performed. The optimized geometries are found to be non-planar, with intra-molecular hydrogen bonding interactions within the pyrazine ring. The HOMO–LUMO energy analysis showed that in most cases the piperazine ring contributes more to the HOMO than the other moieties in the molecule, whereas the LUMO is stabilised by the pyrazine ring. The chemical potential from the computed global descriptors suggests that all molecules are stable. The ESPs of molecules reveal the active sites for nucleophilic and electrophilic attacks. ADMET studies show that all molecules have good pharmacological properties and are nontoxic (class 4). The predicted pIC_50_ values show that the derivatives studied are better than the already-existing drug favipiravir. The BE from molecular docking studies in almost all cases correlates with the pIC_50_ value predicted. The pIC_50_ values correlate with the H–L gap and the BE of the molecule with protein. It was also noted that the number of rotatable bonds in the molecule increases the BE as well as the pIC_50_ value. This study suggests the tuning of the chemical properties of piperazine-substituted favipiravir derivatives to be a promising tool in designing inhibitors for NiV.

## Supplementary Information

Below is the link to the electronic supplementary material.

Supplementary file1 (PDF 2048 kb)
