# Supplementary material for: Piperazine-substituted derivatives of favipiravir for Nipah virus inhibition: What do in silico studies unravel?
Source: SN Appl Sci. 2021 Jan 11;3(1):110. doi: 10.1007/s42452-020-04051-9 (PMC7799160; doi:10.1007/s42452-020-04051-9)
Supplement: Supplementary file 1 — Supplementary file1 (PDF 2048 kb) [file 42452_2020_4051_MOESM1_ESM.pdf]

Supplementary Material

Fig. S1. 3D view of the binding conformations of molecule at the active site of the protein

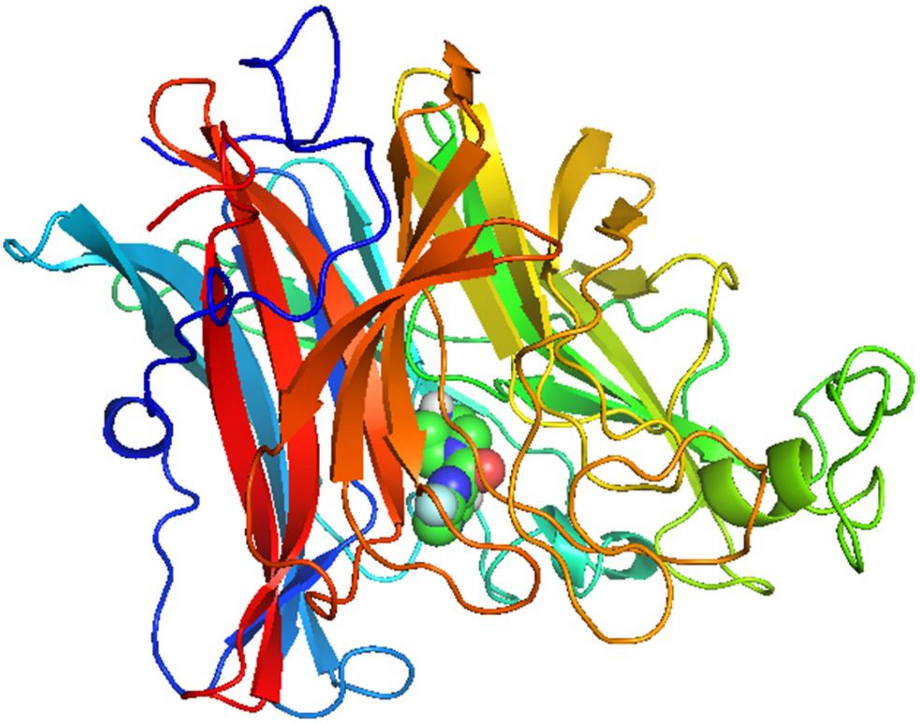

F1

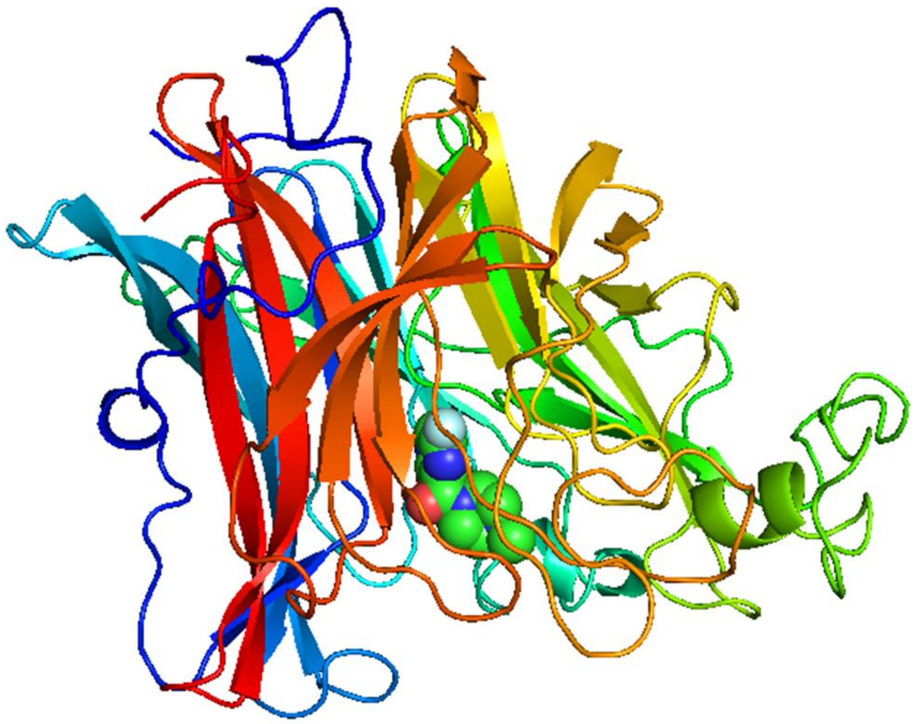

F2

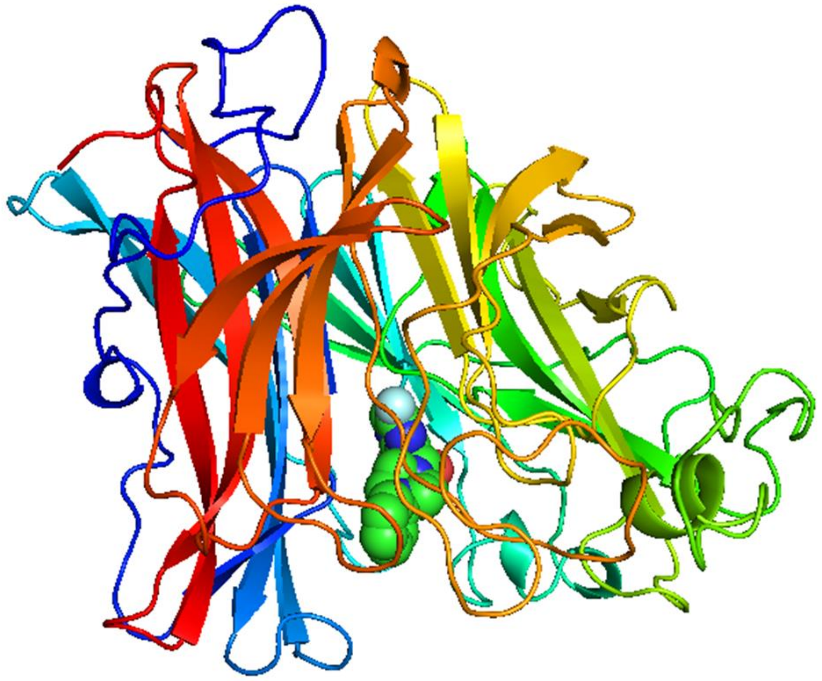

F3

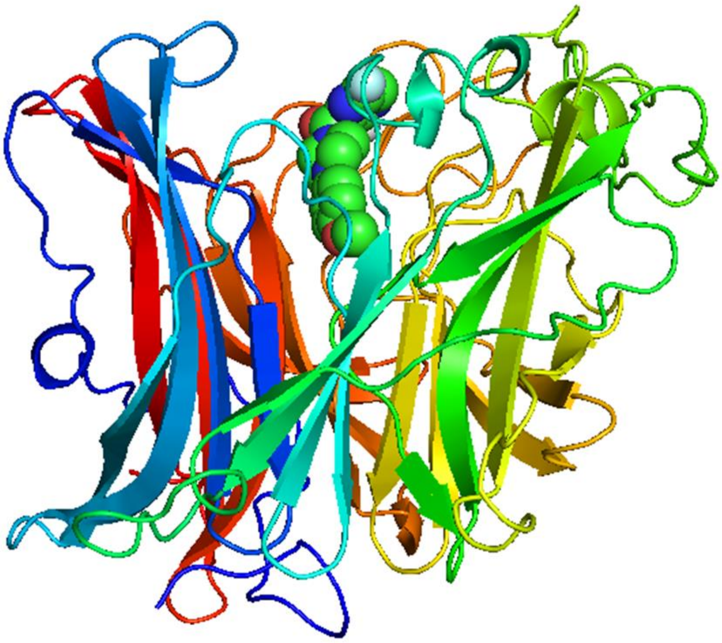

F4

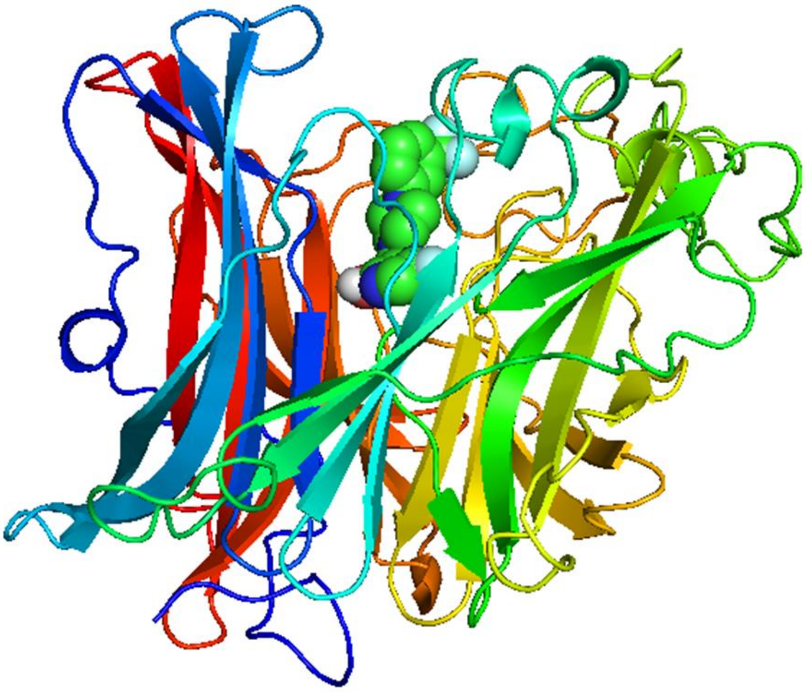

F5

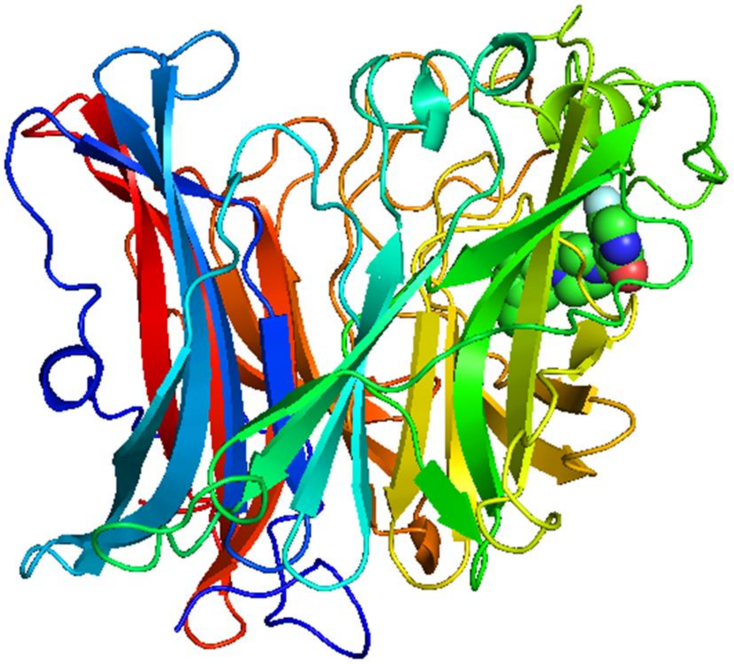

F6

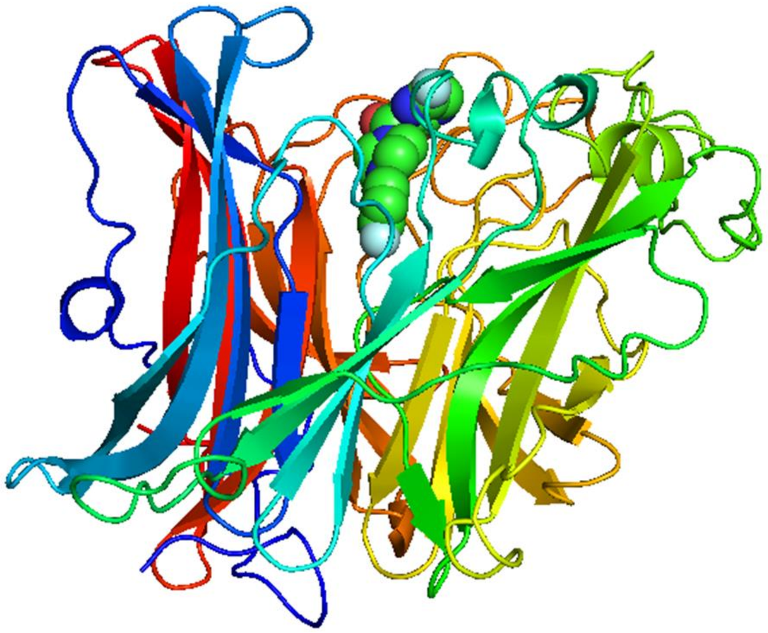

F7

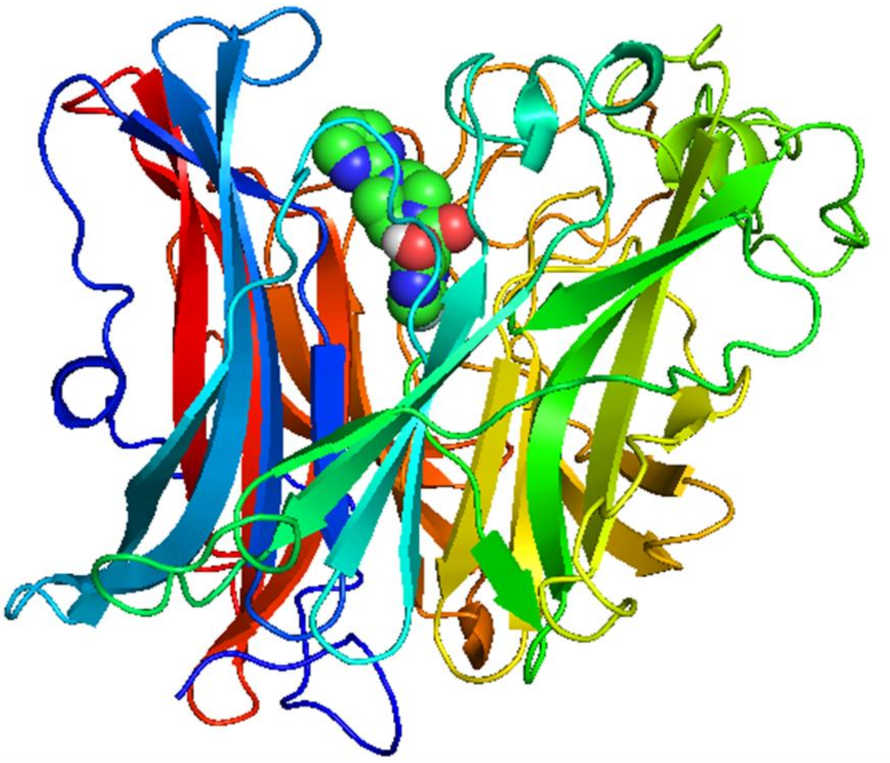

F8

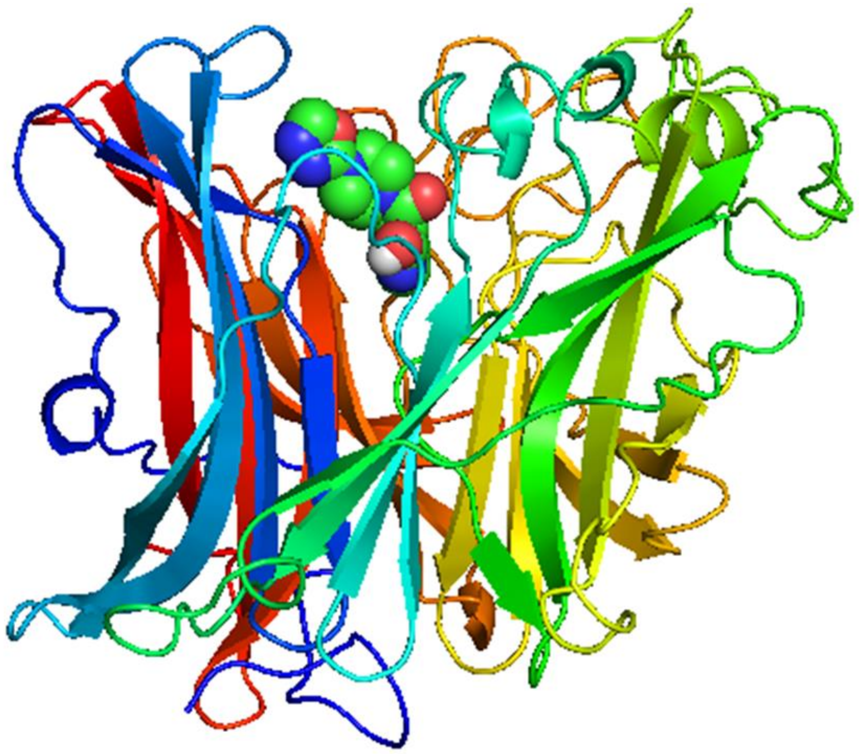

F9

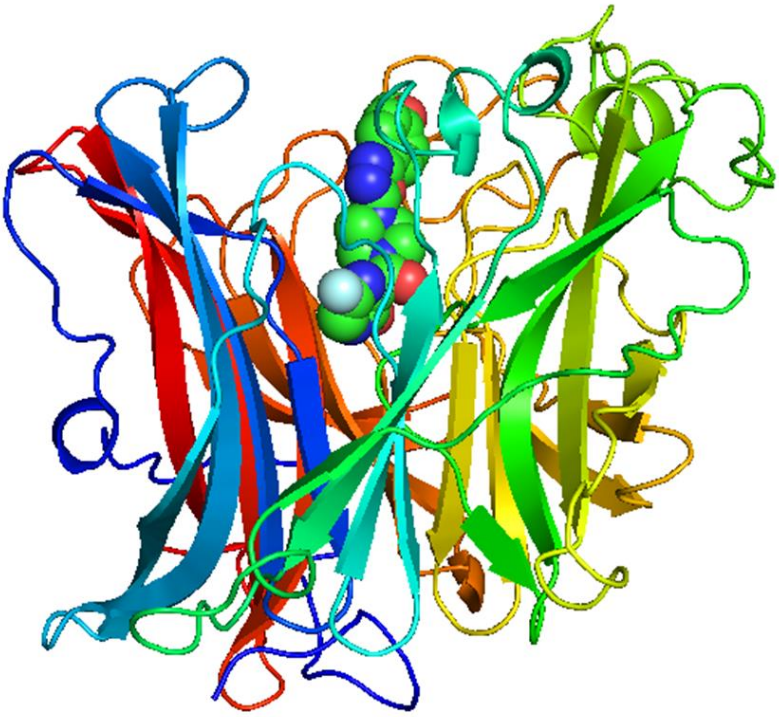

F13

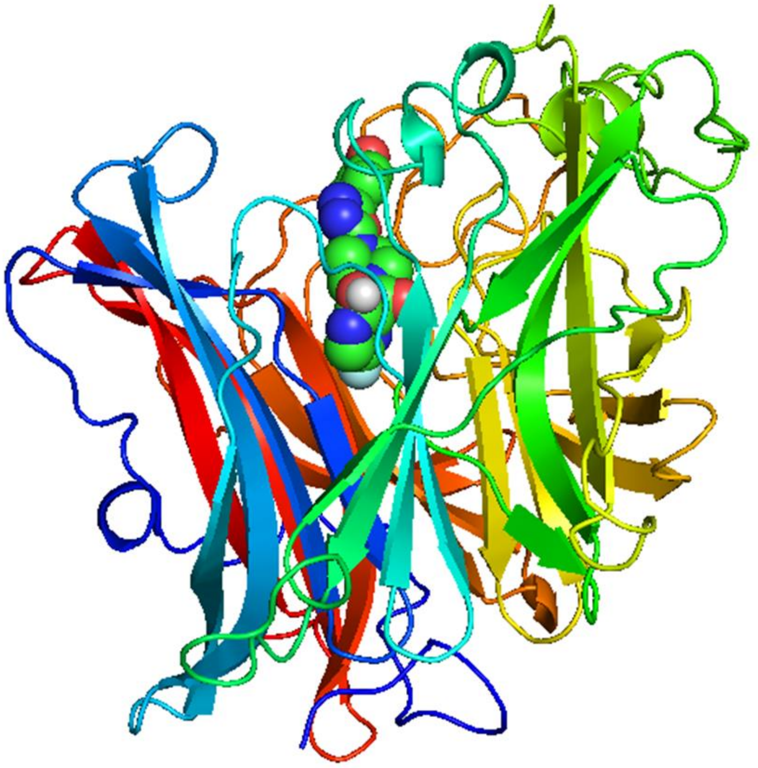

F14

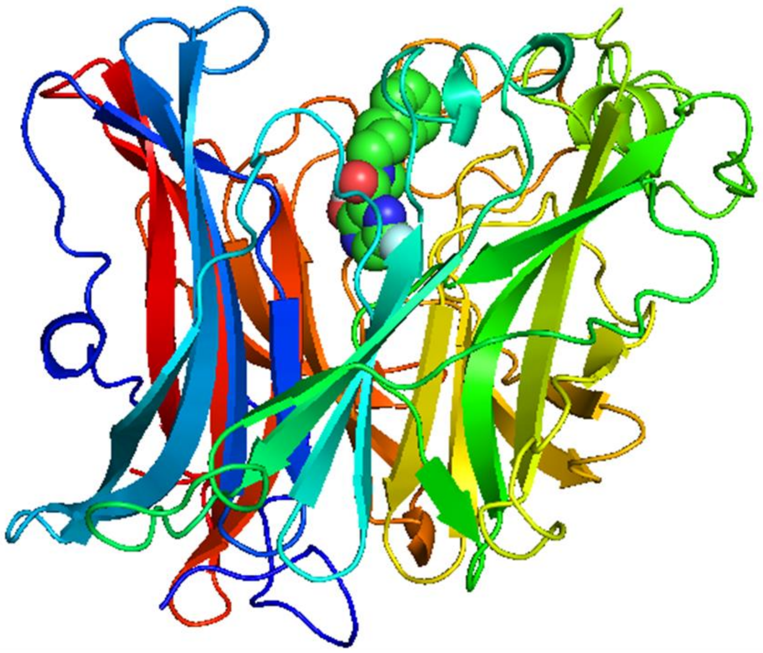

F15

Fig S2. Interactions made by the molecules with the targeted protein (3D11) analysed using Ligplot

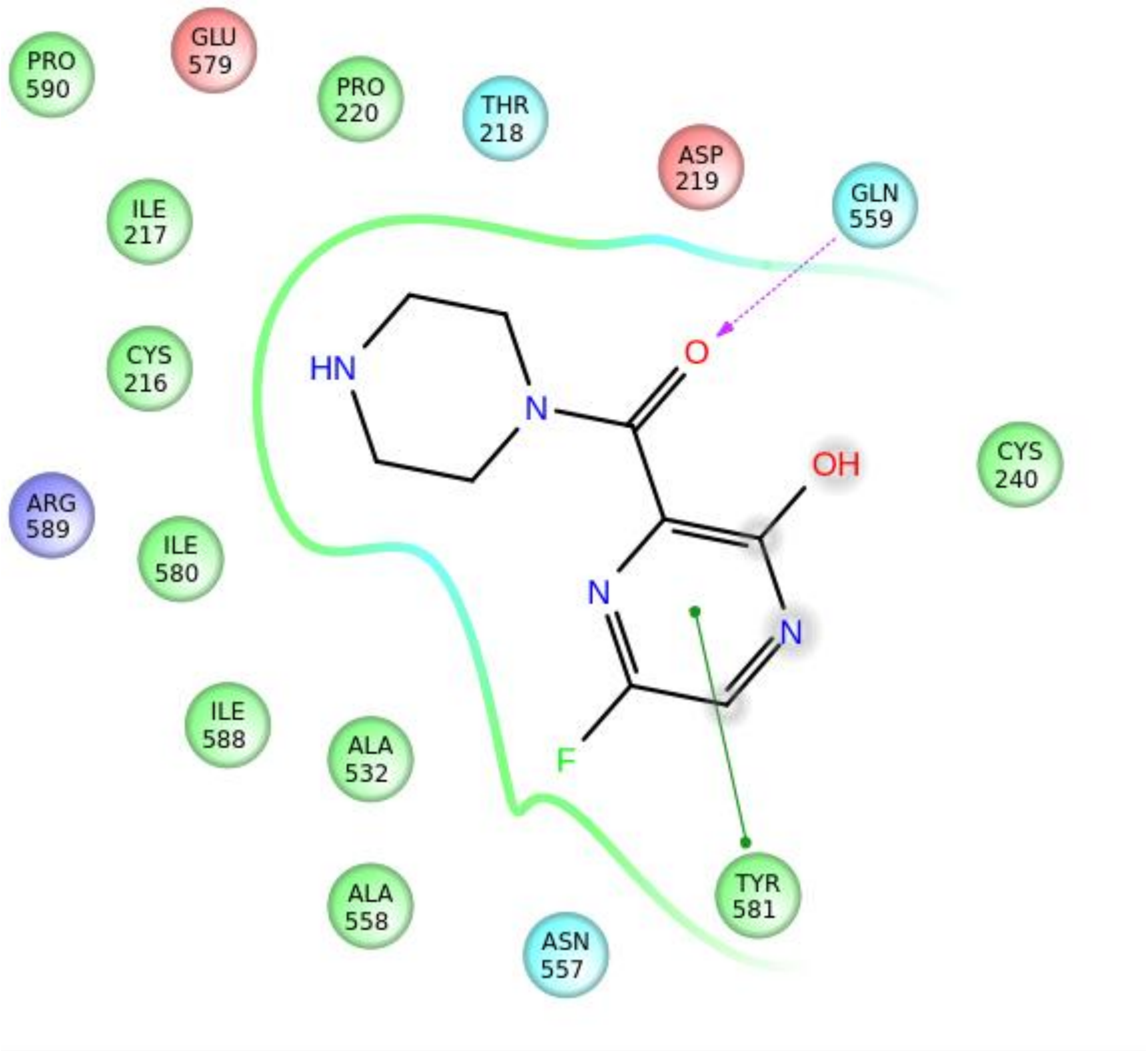

F1

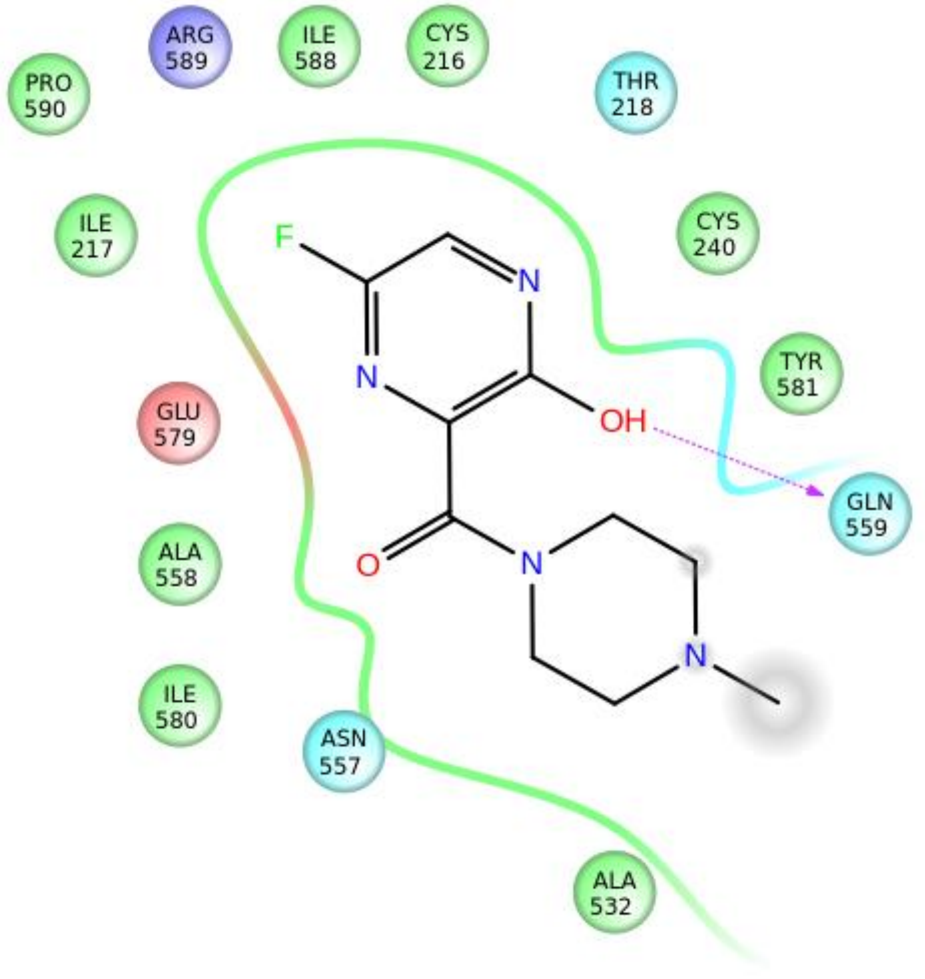
PRO 590
ARG 589
ILE 588
CYS 216
THR 218
ILE 217
CYS 240
TYR 581
GLU 579
GLN 559
ALA 558
ILE 580
ASN 557
ALA 532
F
N
N
OH
O
N
N

F2

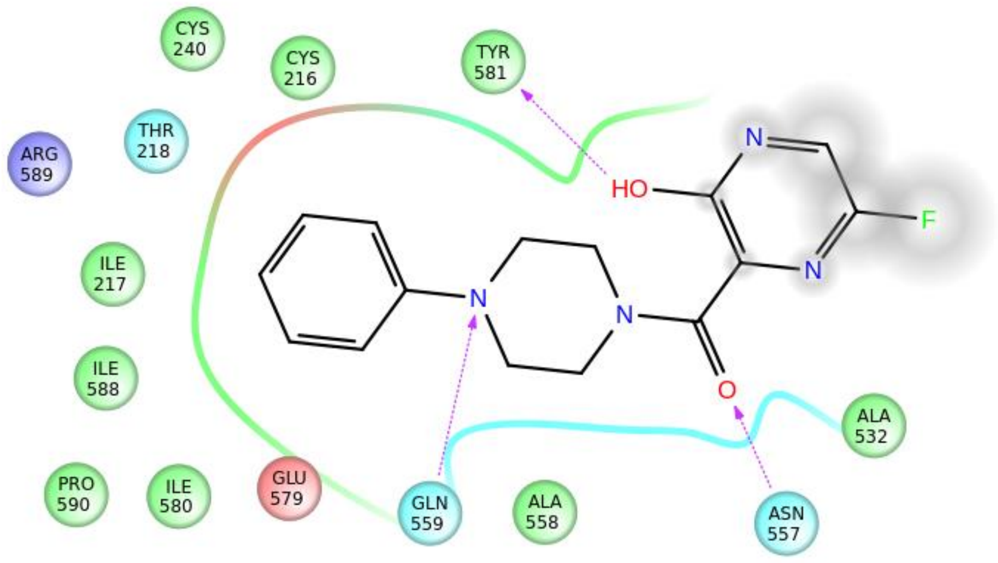
CYS
240
CYS
216
TYR
581
ARG
589
THR
218
HO
N
F
N
ILE
217
N
N
O
ILE
588
ALA
532
PRO
590
ILE
580
GLU
579
GLN
559
ALA
558
ASN
557

F3

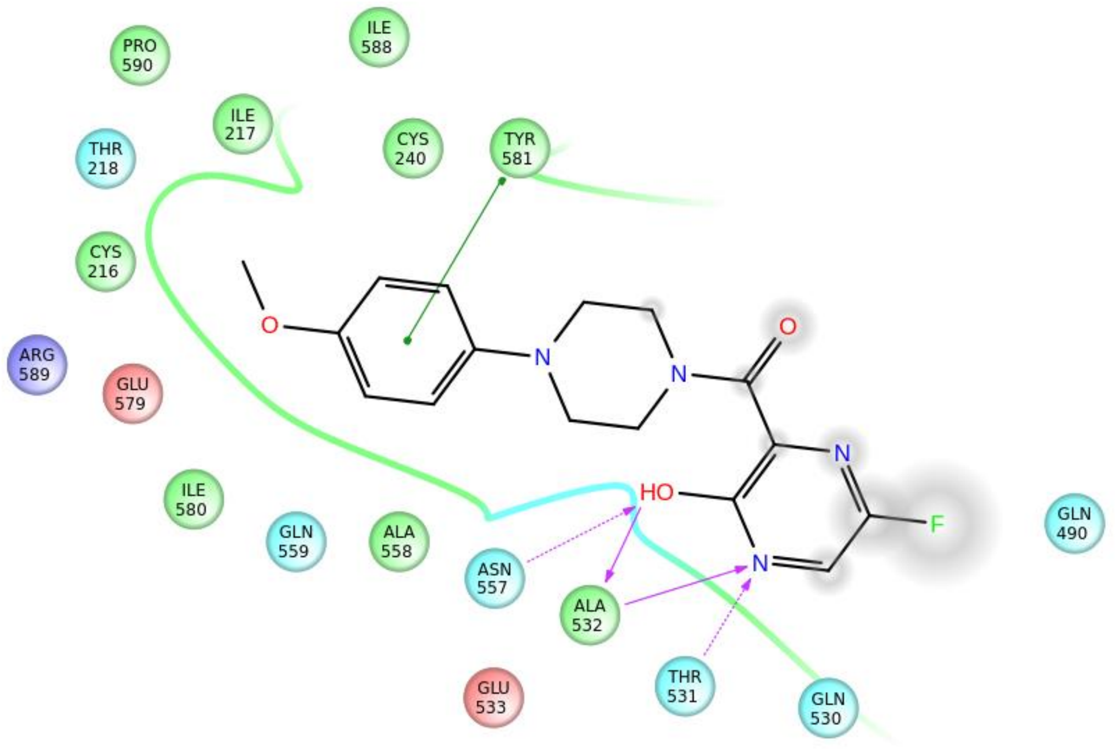

PRO 590
ILE 588
ILE 217
CYS 240
TYR 581
THR 218
CYS 216
ARG 589
GLU 579
ILE 580
GLN 559
ALA 558
ASN 557
ALA 532
GLU 533
THR 531
GLN 530
GLN 490
O
N
N
O
N
HO
F
N

F4

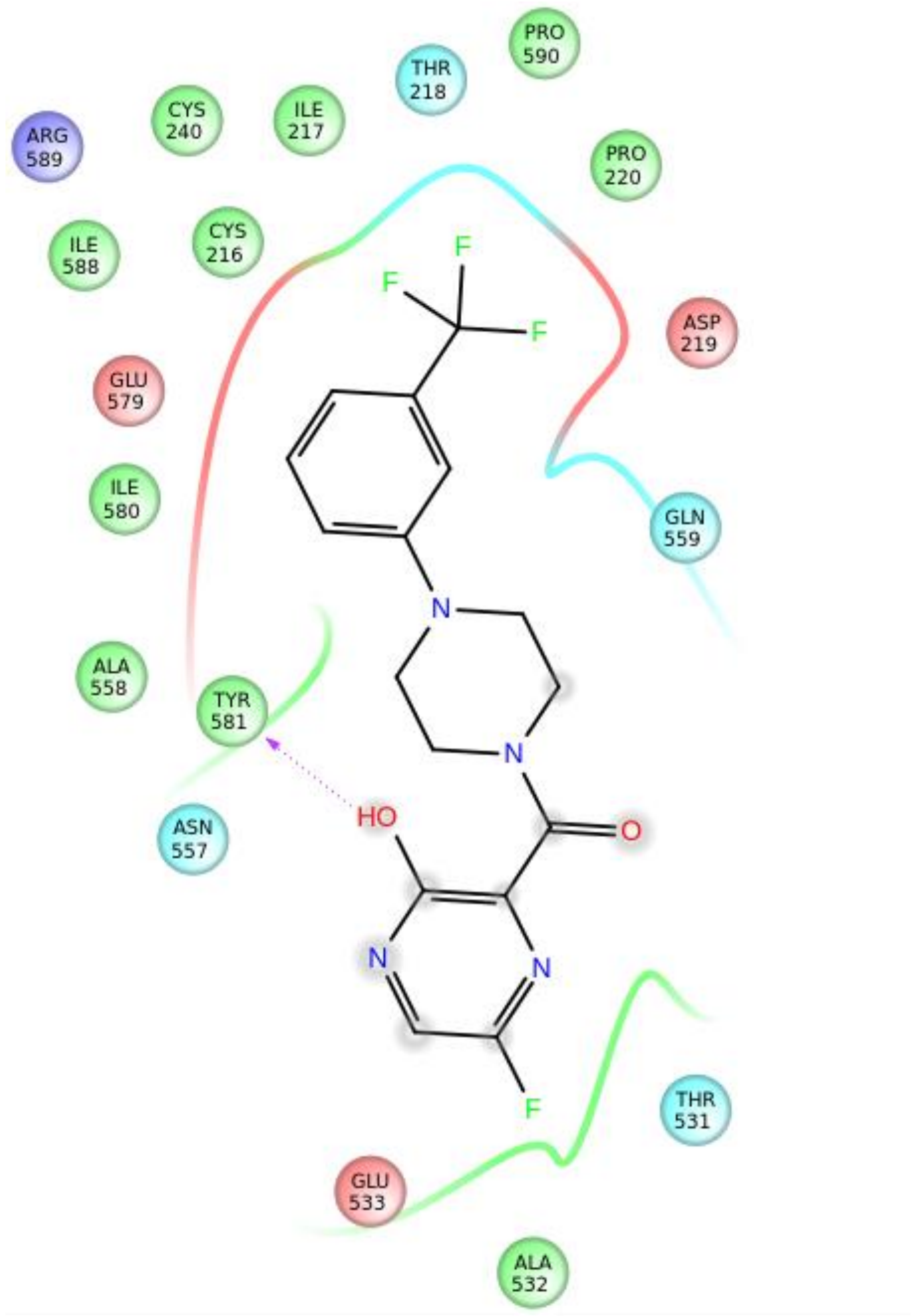
PRO 590
THR 218
CYS 240
ILE 217
ARG 589
PRO 220
ILE 588
CYS 216
F
F
F
ASP 219
GLU 579
ILE 580
GLN 559
N
ALA 558
TYR 581
N
HO
O
ASN 557
N
N
F
THR 531
GLU 533
ALA 532

F5

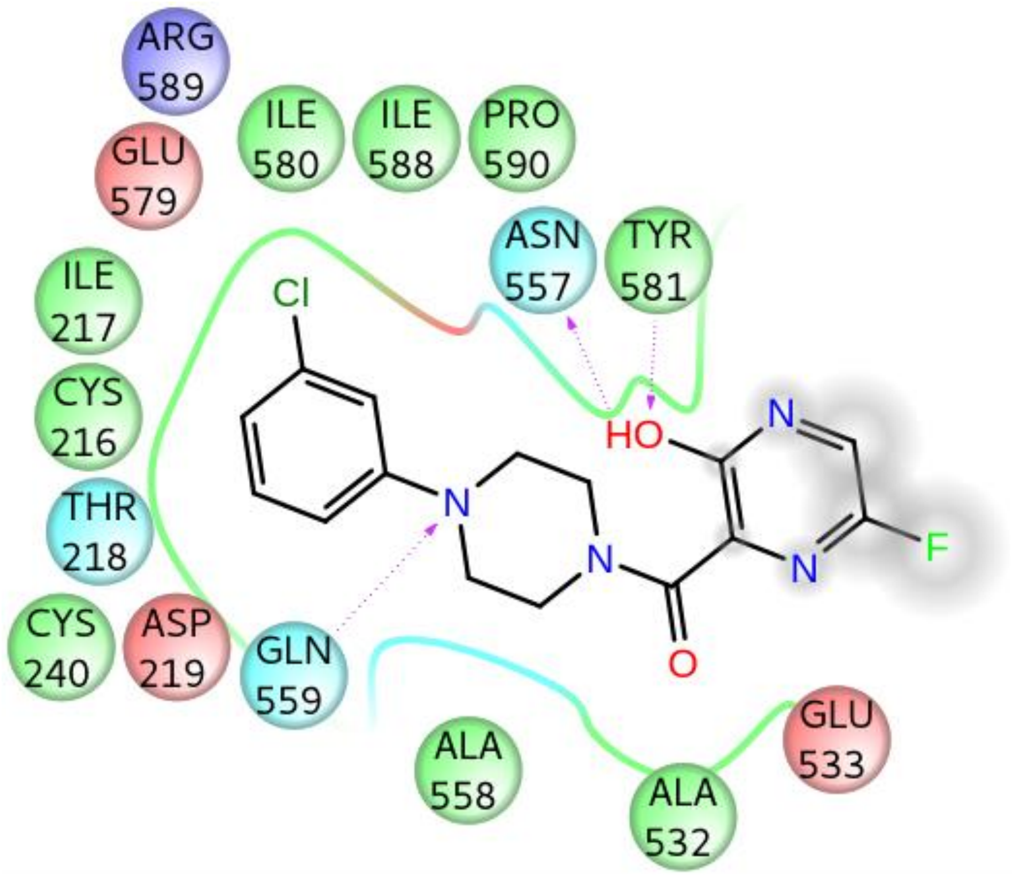
ARG 589
GLU 579
ILE 580
ILE 588
PRO 590
ILE 217
CYS 216
THR 218
CYS 240
ASP 219
GLN 559
ASN 557
TYR 581
ALA 558
ALA 532
GLU 533
Cl
N
N
HO
N
N
F
O

F6

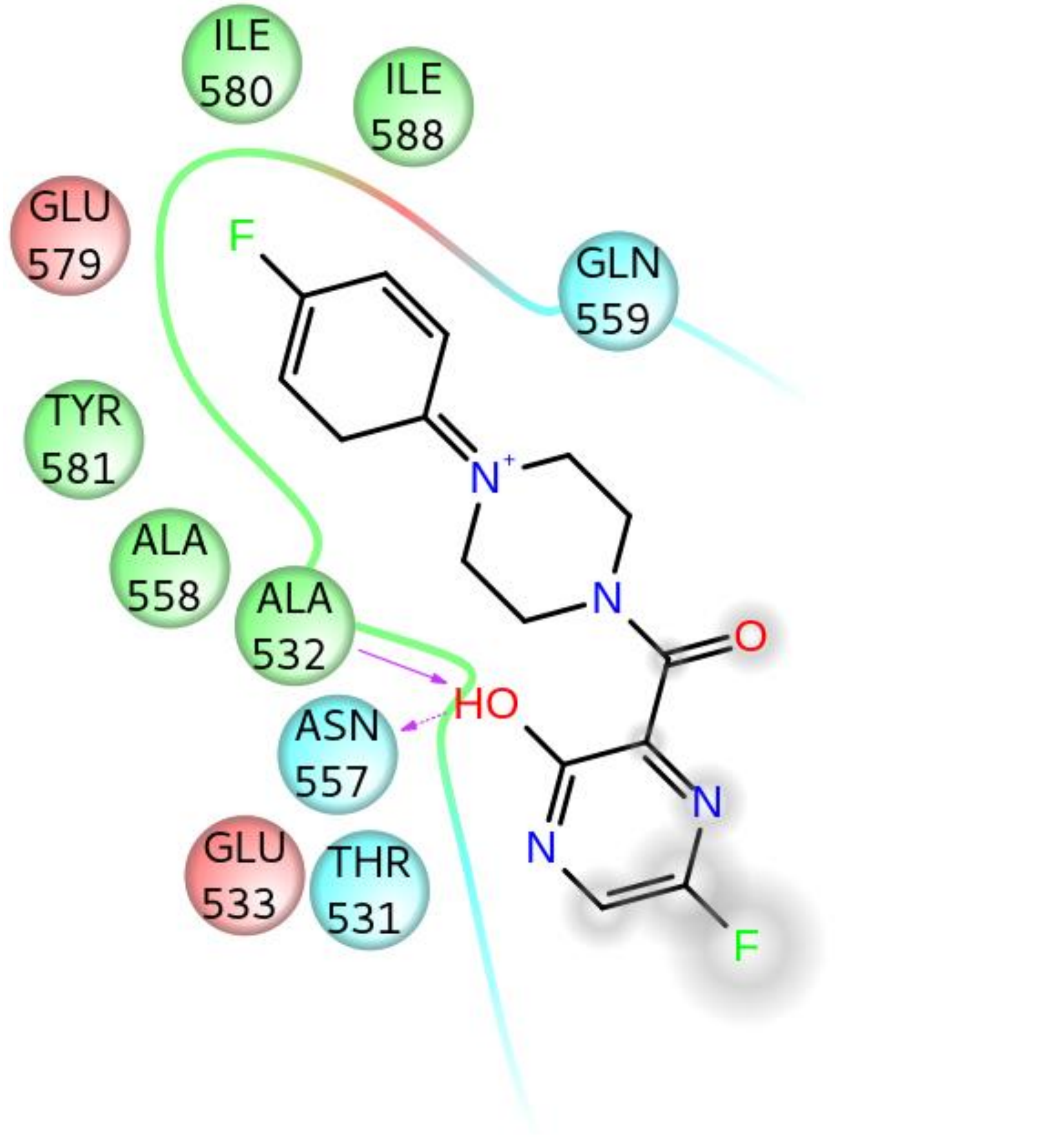
ILE 580
ILE 588
GLU 579
GLN 559
TYR 581
ALA 558
ALA 532
ASN 557
GLU 533
THR 531
F
N+
N
O
HO
N
N
F

F7

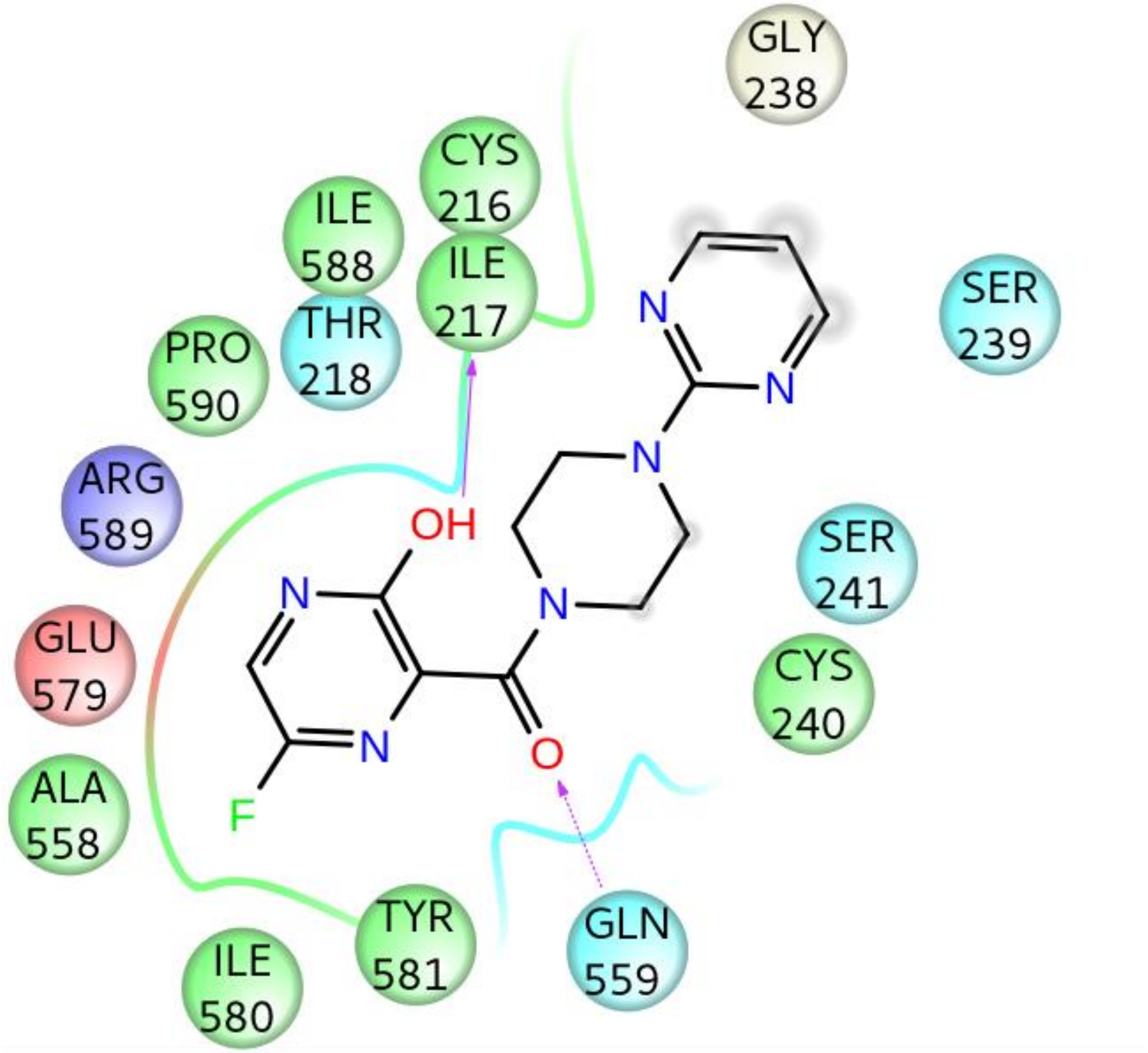
GLY
238
CYS
216
ILE
588
ILE
217
SER
239
THR
218
PRO
590
N
N
N
ARG
589
OH
SER
241
N
N
GLU
579
CYS
240
N
O
ALA
558
F
TYR
581
GLN
559
ILE
580

F8

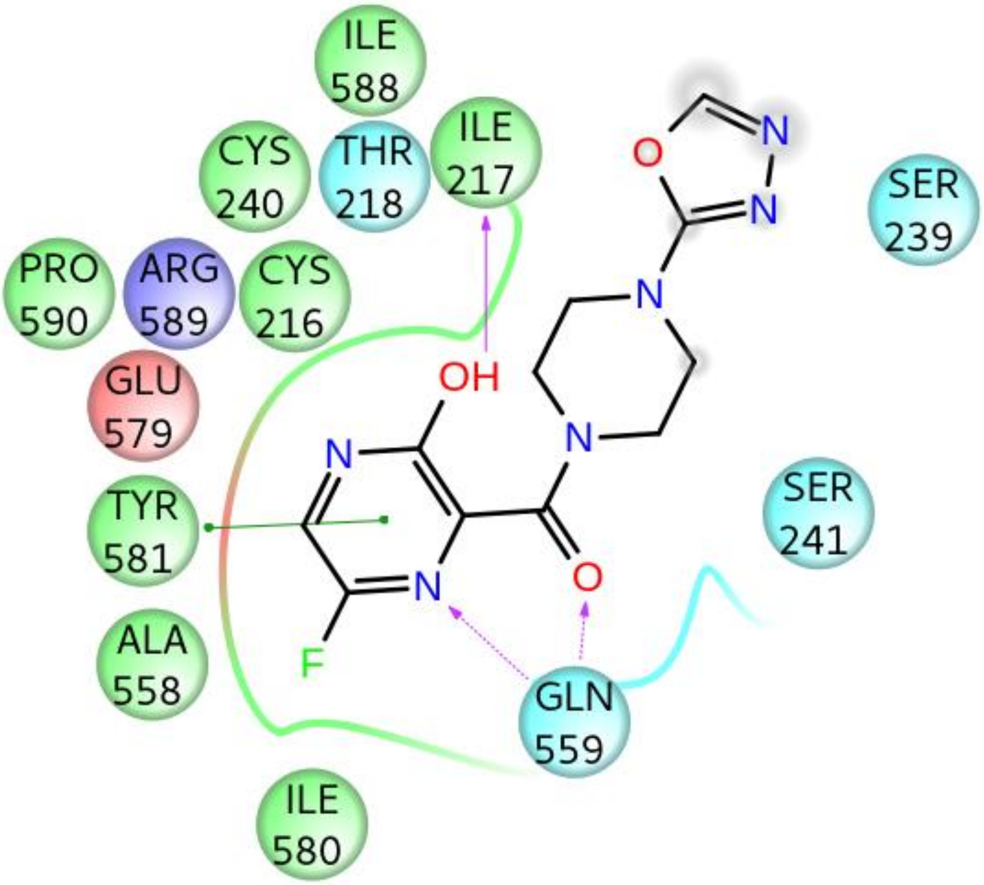
ILE
588
CYS
240
THR
218
ILE
217
PRO
590
ARG
589
CYS
216
GLU
579
TYR
581
ALA
558
ILE
580
GLN
559
SER
239
SER
241
OH
N
N
F
O
N
N
O
N
N

F9

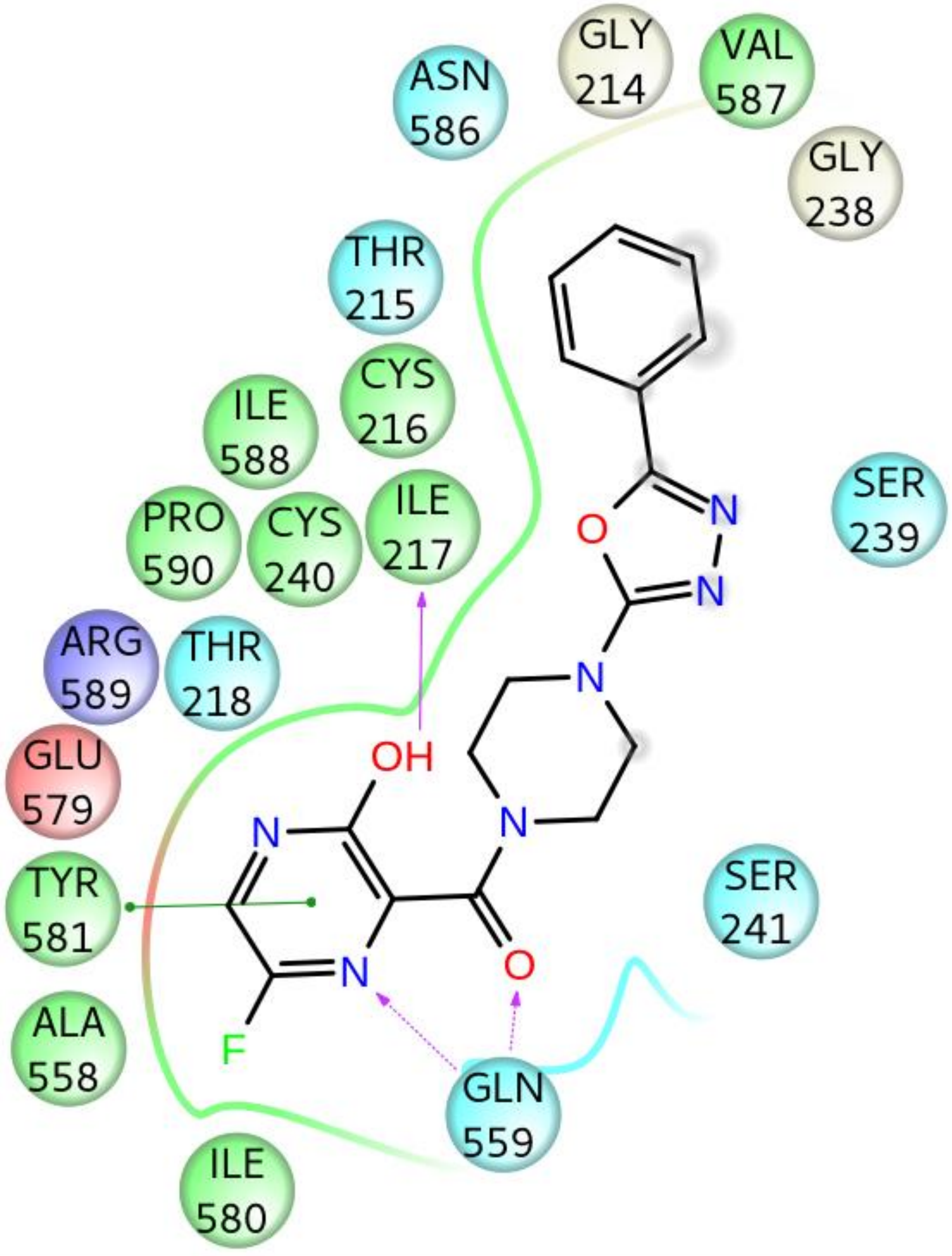
GLY 214
VAL 587
ASN 586
GLY 238
THR 215
CYS 216
ILE 588
SER 239
PRO 590
CYS 240
ILE 217
ARG 589
THR 218
GLU 579
TYR 581
SER 241
ALA 558
GLN 559
ILE 580
OH
N
O
F

F10

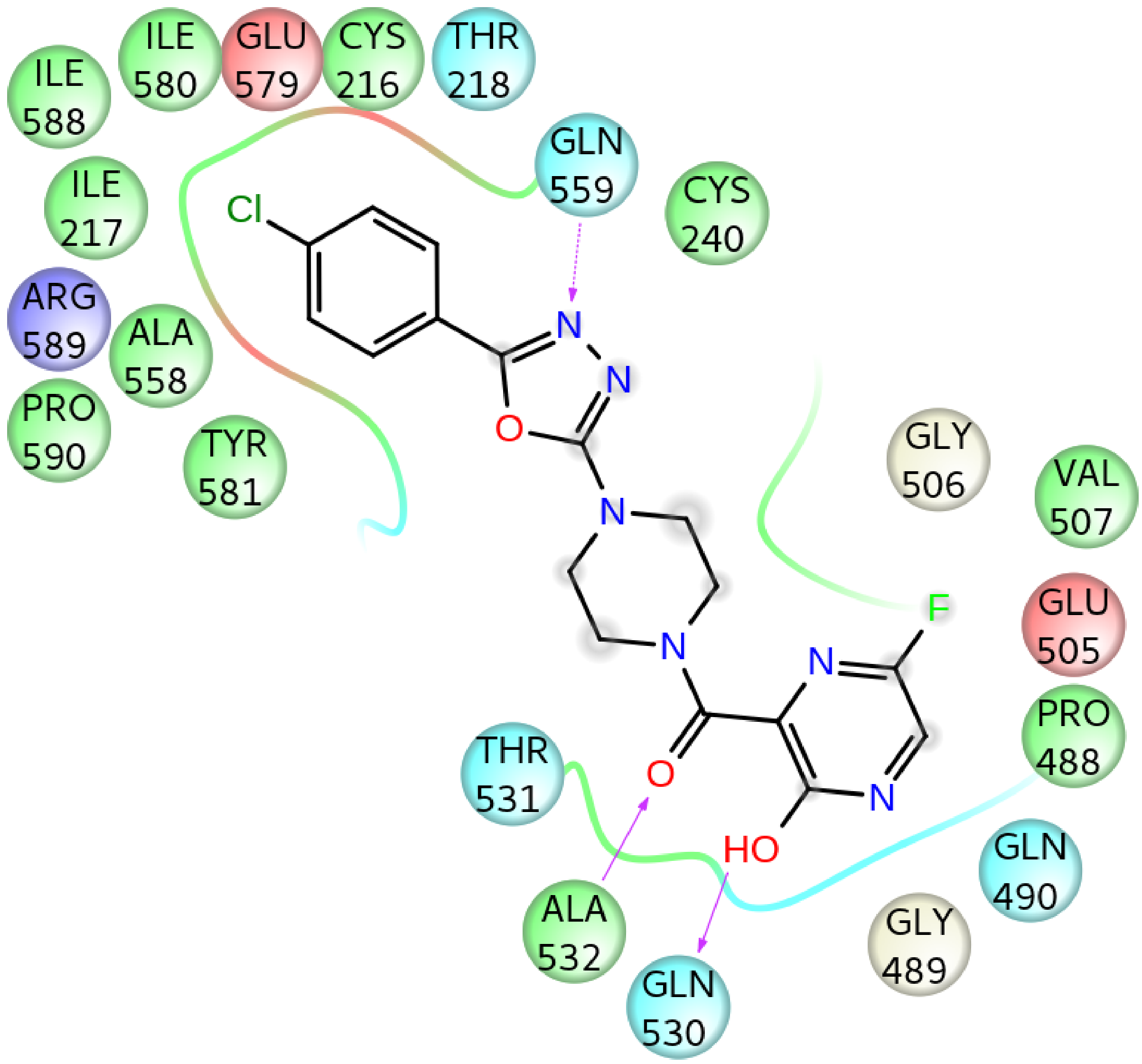
ILE 588
ILE 580
GLU 579
CYS 216
THR 218
GLN 559
CYS 240
ILE 217
Cl
ARG 589
ALA 558
PRO 590
TYR 581
N
N
O
N
GLY 506
VAL 507
F
GLU 505
N
N
PRO 488
THR 531
O
N
HO
GLN 490
ALA 532
GLY 489
GLN 530

F11

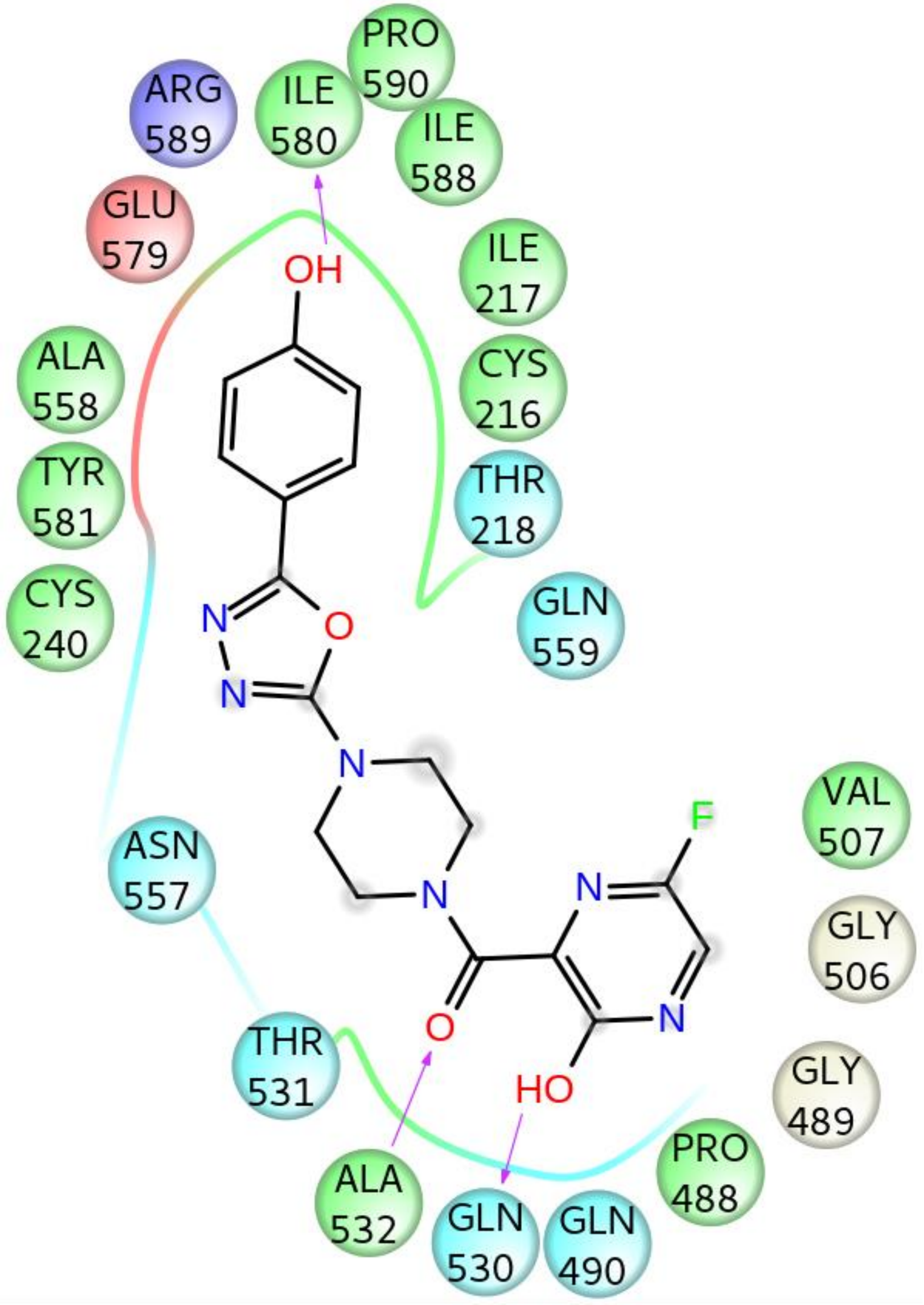
PRO
590
ARG
589
ILE
580
ILE
588
GLU
579
OH
ILE
217
ALA
558
CYS
216
TYR
581
THR
218
CYS
240
N
O
GLN
559
N
N
VAL
507
F
ASN
557
N
N
GLY
506
O
N
THR
531
HO
GLY
489
PRO
488
ALA
532
GLN
530
GLN
490

F12

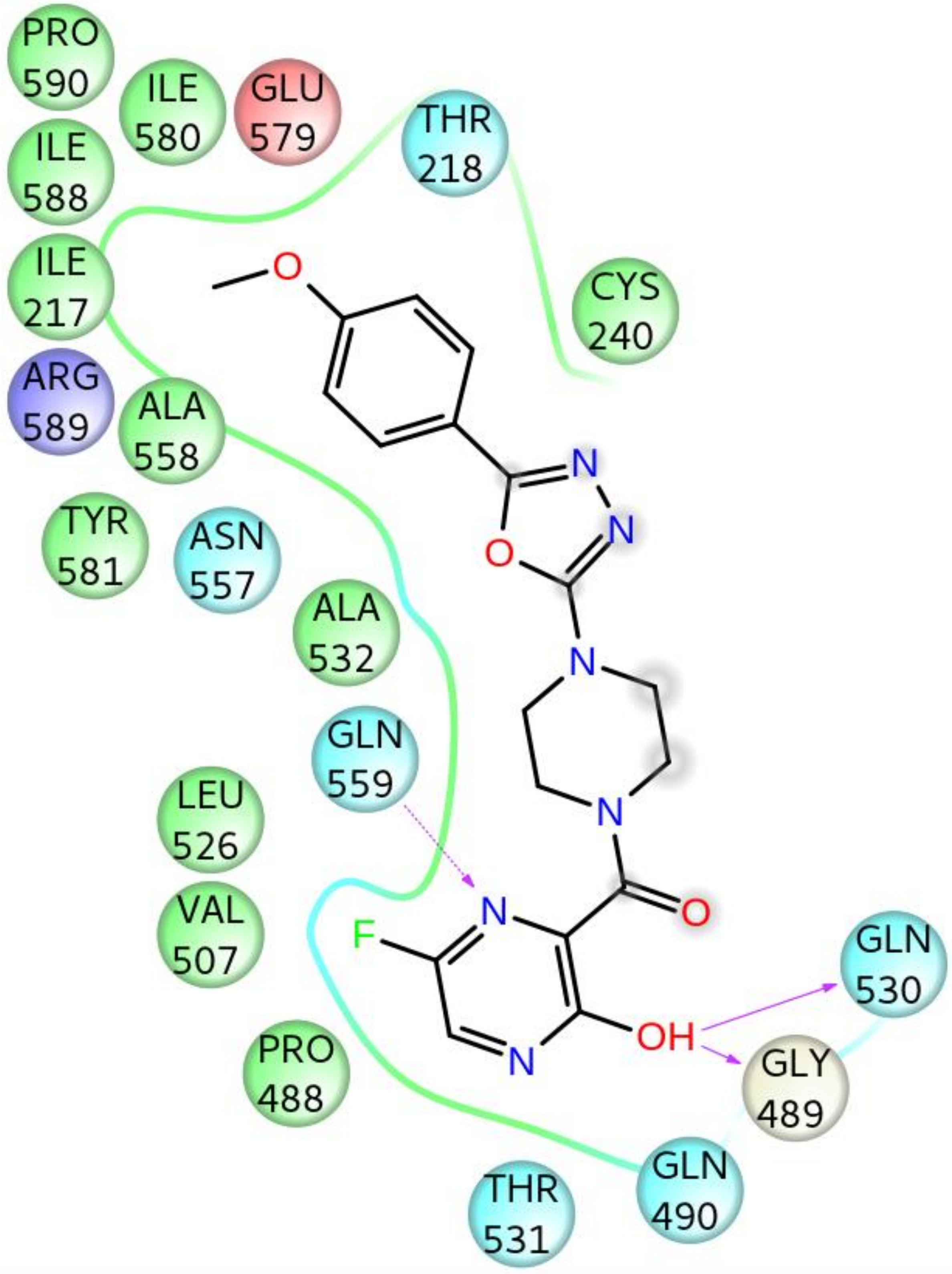
PRO
590
ILE
580
GLU
579
THR
218
ILE
588
ILE
217
CYS
240
ARG
589
ALA
558
TYR
581
ASN
557
ALA
532
GLN
559
LEU
526
VAL
507
GLN
530
GLY
489
PRO
488
GLN
490
THR
531
O
N
N
O
N
N
F
N
O
N
OH

F13

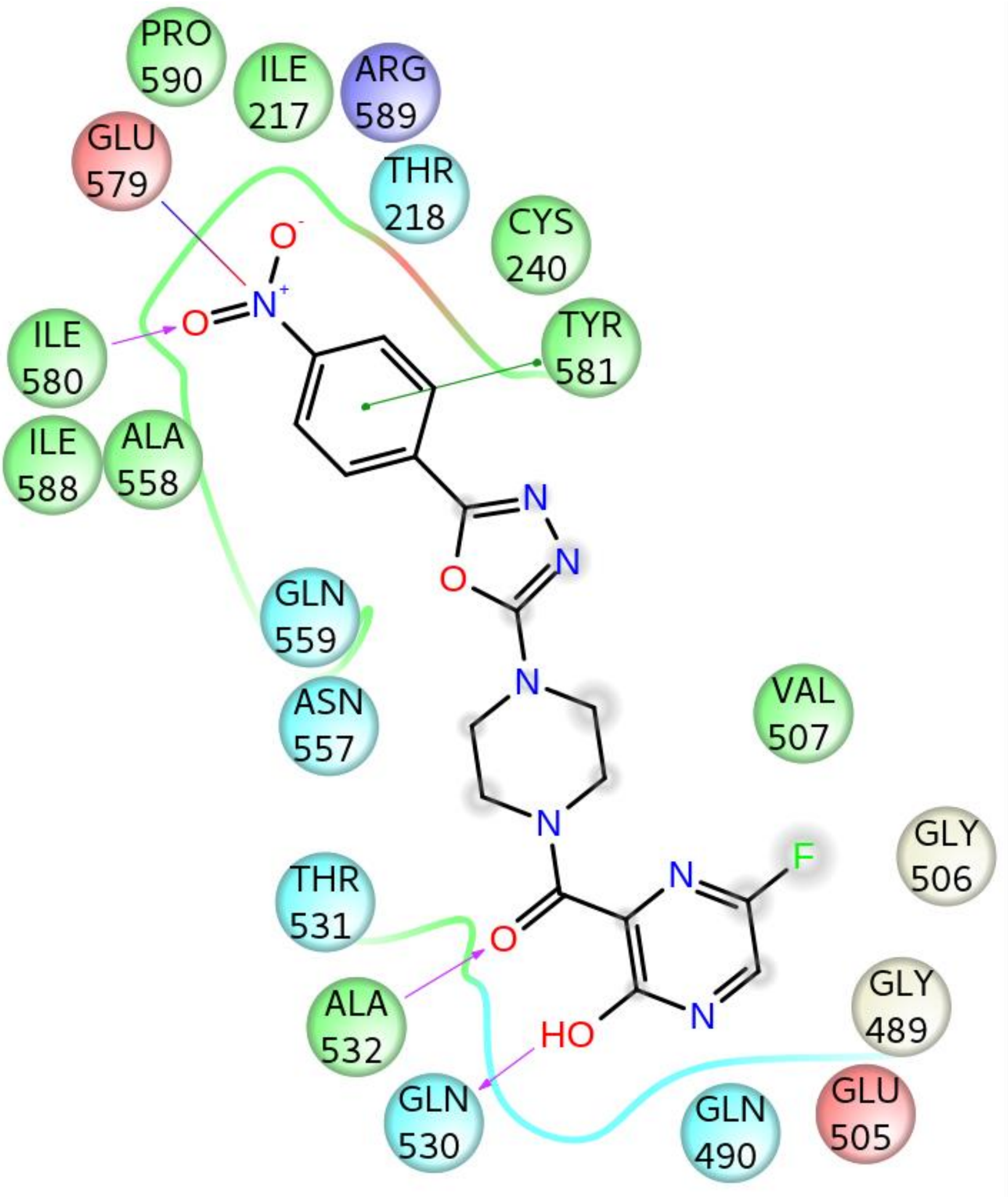
PRO 590
ILE 217
ARG 589
GLU 579
THR 218
CYS 240
O-
N+
ILE 580
O
TYR 581
ILE 588
ALA 558
N
N
O
GLN 559
N
ASN 557
VAL 507
N
GLY 506
F
N
THR 531
O
GLY 489
N
ALA 532
HO
GLN 530
GLN 490
GLU 505

F14
